# Heterogeneously catalyzed supramolecular polymerization: essential roles of nucleation and fragmentation-induced autocatalysis in chiral transfer†

**DOI:** 10.1039/d4sc07894b

**Published:** 2025-02-18

**Authors:** Peichen Shi, Ganyu Chen, Qiang Chen, Huiting Wu, Suixu Li, Xiaoyu Cao, Liulin Yang, Zhongqun Tian

**Affiliations:** a State Key Laboratory of Physical Chemistry of Solid Surface, Key Laboratory of Chemical Biology of Fujian Province, Collaborative Innovation Center of Chemistry for Energy Materials (iChEM), Innovation Laboratory for Sciences and Technologies of Energy Materials of Fujian Province (IKKEM), Department of Chemistry, College of Chemistry and Chemical Engineering, Xiamen University Xiamen 361005 P. R. China gychen321@163.com xcao@xmu.edu.cn llyang@xmu.edu.cn zqtian@xmu.edu.cn

## Abstract

The complexity of multi-component molecular assembly demands precise control strategies to enhance both efficiency and selectivity. Heterogeneous nucleation and the autocatalytic secondary pathway, as key regulatory strategies, have attracted widespread attention for their crucial roles in crystal growth and amyloid protein aggregation. Here, we apply a heterogeneous nucleation strategy to supramolecular polymer systems and report the first direct observation of surface-enrichment-induced primary nucleation and a spontaneous fragmentation-driven autocatalytic secondary process. A heterogeneous nucleating agent promotes primary nucleation, facilitating supramolecular chiral induction. The resulting chiral polymers undergo a catalytic cycle of fragmentation and re-growth at their termini, with the fragments also acting as seeds for nucleation and growth. These pathways play a crucial role in the polymerization process and are essential for chiral transfer and asymmetry amplification, enabling the achievement of maximum enantioselectivity with as little as 0.5% molar equivalent of the heterogeneous nucleating agent. Furthermore, we reveal the existence of an optimal equivalent in their catalytic kinetics, arising from a surface assembly mechanism. In this mechanism, monomers adsorbed on the surface of the heterogeneous nucleating agent assemble with those in solution, rather than through surface diffusion and assembly. This process resembles the surface-catalyzed Eley–Rideal mechanism. Our study highlights the potential of heterogeneous nucleation as an effective strategy for controlling supramolecular polymerization and offers new insights into its underlying mechanism.

## Abstract 2

We unveil spontaneous fragmentation-induced autocatalysis in heterogeneously catalyzed supramolecular polymerization, where nucleating agents serve as catalysts, driving efficient chiral induction and acceleration at an optimal catalytic equivalent.

## Graphical abstract

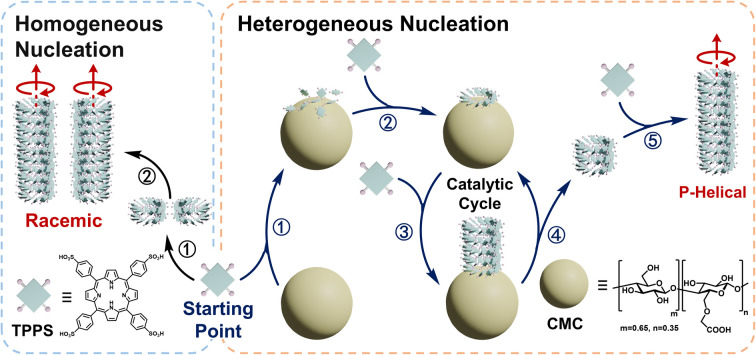

## Introduction

Supramolecular polymerization, observed in both natural and synthetic systems, involves the polymerization of monomers through non-covalent interactions, exhibiting dynamic and reversible behavior.^1–3^ While prevalent in biological systems such as actin filaments^4^ and tubulin,^5^ achieving the precise control evident in these systems remains challenging in synthetic supramolecular polymers. As a result, researchers are increasingly focusing on kinetically regulating supramolecular polymer systems by shifting from traditional single-step self-assembly to multistep noncovalent synthetic strategies.^6–13^ Various approaches—such as template utilization,^14–16^ seed incorporation,^17–19^ artificial molecular chaperones,^20–22^ pH^23^ and temperature modulation,^24^ and application of external fields such as light,^25^ electric,^26,27^ and flow^28,29^—have been employed to accelerate assembly processes and regulate the product distribution.

Nucleation can be significantly accelerated at phase interfaces or when additional particles are introduced into solutions, a phenomenon known as heterogeneous nucleation. In biological systems, heterogeneous nucleation plays a crucial role in protein aggregation. For instance, studies have shown that lipid membranes induce the nucleation and aggregation of Aβ proteins on their surface.^30^ In artificial supramolecular polymerization systems, researchers have explored chiral templates, such as biomacromolecules like peptides,^31^ DNA,^32–34^ and polysaccharides,^35,36^ to induce chirality in achiral monomers. These chiral templates function similarly to heterogeneous nucleating agents, which are typically regarded as co-assembling with the substrate. However, the potential of heterogeneous nucleation to further enable catalytic cycles and a secondary pathway in supramolecular polymerization remains largely unexplored. These mechanisms, akin to the role of a catalyst, could significantly enhance chiral induction effects with minimal heterogeneous nucleating agents, while allowing chiral inducers to avoid interfering with the assembly and morphology of the final products. Furthermore, the mechanisms of surface assembly in heterogeneous nucleation systems require more comprehensive quantitative analysis.

**Scheme 1:**
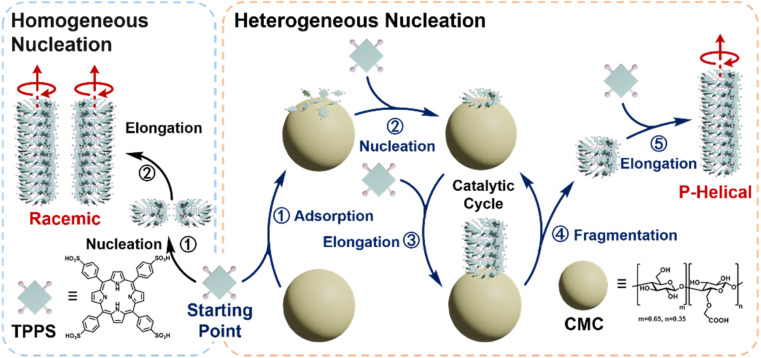
Schematic representation of the competitive pathways of TPPS homogeneous nucleation and heterogeneous nucleation with CMC as the nucleating agent.

Herein, we demonstrate the critical role of the fragmentation-induced secondary pathway in promoting chiral transfer and asymmetry amplification in heterogeneously catalyzed supramolecular polymerization (Scheme 1). We report that the polysaccharide carboxymethyl cellulose (CMC) acts as a heterogeneous nucleating agent, effectively catalyzing the supramolecular chiral polymerization of achiral *meso*-tetraphenylsulfonato porphyrin (TPPS) monomers. CMC promotes the formation of TPPS supramolecular polymers with a predominant *p*-helical chiral form, achieving maximum enantioselectivity even at molar equivalents as low as 0.5% (calculated based on polymer repeating units relative to TPPS monomers). Real-time super-resolution fluorescence microscopy reveals a surface enrichment-induced primary nucleation and a substantial spontaneous fragmentation-induced secondary pathway, which together account for the high efficiency of CMC in chiral transfer and asymmetry amplification. Structure–function relationship analysis indicates that flexible side chains with carboxyl end groups and high degrees of polymerization enhance the functionality of CMC as an efficient chiral heterogeneous nucleating agent. Furthermore, kinetic analysis reveals an optimal equivalent in the catalytic process, attributed to the assembly of TPPS monomers on the CMC surface with those in solution, rather than through surface diffusion, akin to the Eley–Rideal mechanism. This study reveals the intrinsic catalytic properties of heterogeneous nucleating agents in controlling supramolecular polymerization, with a focus on regulating assembly rates and selecting pathways to enhance chiral transfer and asymmetry amplification.

## Results and discussion

Carboxymethyl cellulose (CMC) was selected as a potential heterogeneous nucleating agent for several reasons: (1) CMC has an approximately spherical morphology in acidic aqueous solution (pH = 1) with a diameter of approximately 50 nm (Fig. S11†), thus providing a substantial specific surface area conducive to the adsorption of TPPS monomers and the induction of nucleation. (2) Under the assembly conditions of TPPS, the carboxyl groups of CMC are protonated, and are anticipated to form double hydrogen bonds with the sulfonic acid groups of TPPS, thus pre-organizing TPPS onto the surface with a high local concentration. (3) CMC is a readily available chiral polymer,^37^ which could potentially induce the formation of single-handed TPPS supramolecular polymers, consequently enhancing the enantioselectivity of the resulting products.

### CMC accelerates nucleation and enhances enantioselectivity of TPPS supramolecular polymerization

**Fig. 1:**
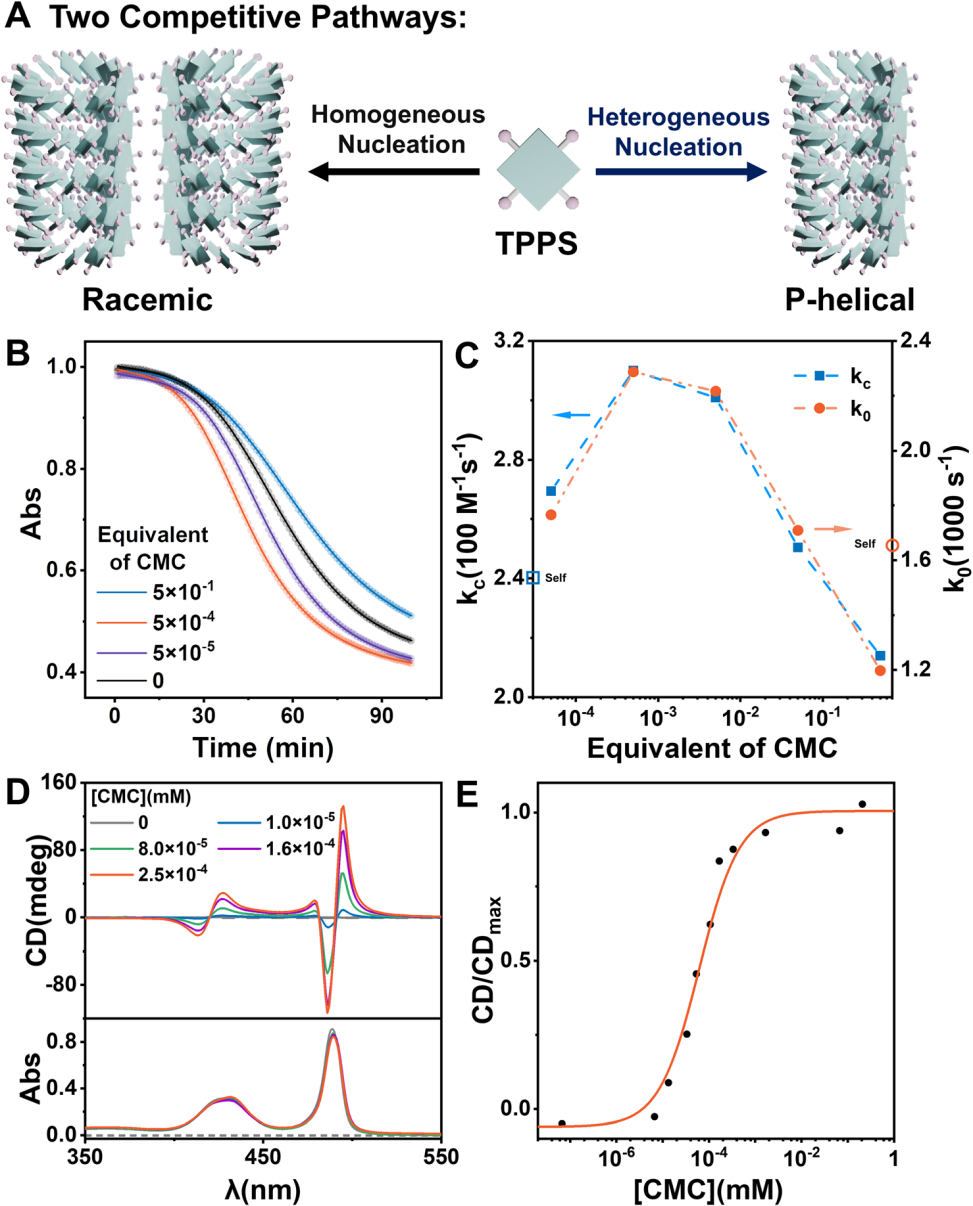
(A) Schematic of the competitive pathways of self-assembly and heterogeneous catalytic assembly. (B) Kinetic curves of the TPPS assembly at different equivalents of CMC. The spectra were obtained by monitoring the absorbance at 434 nm, which show the characteristic peaks of protonated TPPS monomers. The monomer concentration of TPPS was set at 3 μM. (C) *k*_0_ and *k*_c_ obtained from data fitting. (D) CD and UV-vis spectra of TPPS assemblies without and with the addition of different concentrations of CMC. The monomer concentration of TPPS was set at 0.033 mM. (E) The CD/CD_max_ values at 495 nm for TPPS assemblies *versus* different concentrations of CMC.

To investigate the effect of CMC as a heterogeneous nucleating agent, the supramolecular polymerization process of TPPS with varying equivalents of CMC was monitored in real-time. Kinetics data were fitted using a self-similar autocatalytic model^38,39^ where the initial step (*k*_0_) involves the formation of nuclei, alongside a catalytic pathway (*k*_c_) mediated by a porphyrin array (Fig. 1B, S2 and Table S1†). The nucleation rate constant (*k*_0_) and the catalytic rate constant (*k*_c_) were enhanced when the equivalent of CMC (based on repeating units) increased from 0 to 0.05% but subsequently decreased with further increases in equivalent. This indicates that CMC can accelerate nucleation as expected and suggests the presence of an optimal equivalent, with reasons discussed later in the subsequent surface assembly mechanism section (Fig. 5).

Self-assembly of TPPS generates racemic supramolecular polymers, while CMC can induce TPPS to form P-type helical supramolecular polymers, achieving pathway selection for TPPS. Therefore, there exists a competitive relationship between CMC-catalyzed enantioselective supramolecular polymerization of TPPS and the racemic self-assembly of TPPS in solution. CD spectroscopy was employed to evaluate the rate ratio of these two pathways. At low CMC equivalents, the homogeneous self-assembly pathway still dominated (Fig. 1A), resulting in a weak CD signal of the assembly product (Fig. 1D and S3†). With increasing CMC concentrations from 0 to approximately 0.5% molar equivalent, the CD signal gradually increased, and reached a plateau (Fig. 1E, S4 and S24 ESI Section 5.2† for the fitting model). At this point, the heterogeneous catalytic pathway predominated, fully suppressing the self-assembly pathway. When the equivalent of CMC was further increased, it did not further enhance the enantioselectivity. Therefore, the threshold of 0.5% molar equivalent (concentration of 0.16 μM calculated by using repeating units) can be referred as the critical saturated equivalent of CMC (Fig. 1E). This indicates the high efficiency of chiral transfer of CMC compared to previously reported polymers.^35,40–43^

### Chiral transfer was amplified through an autocatalytic cycle of fragmentation and growth

**Fig. 2:**
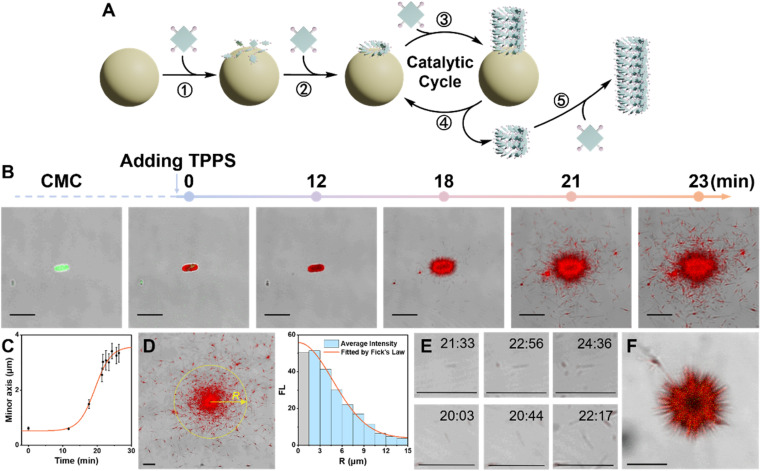
(A) Schematic diagram illustrating primary and fragment-induced secondary processes of TPPS facilitated by CMC. (B) Real-time CLSM overlay images showing the assembly of TPPS on the surface of a CMC aggregate. (C) Statistical analysis of the thickness of the TPPS layer on the CMC aggregate surface over time. (D) Statistical analysis of the fluorescence density of TPPS at various radii around the CMC aggregate, fitted according to Fick's law. (E) CLSM overlay images at various time points depicting the growth process of TPPS fragmented from the CMC aggregate surface. (F) CLSM image displays the longitudinal extension of linear TPPS assemblies growing from the CMC surface in solution. Note: scale bar 5 μm.

To understand how CMC can efficiently induce the enantioselective supramolecular polymerization of TPPS, the assembly process was monitored using multi-channel confocal fluorescence microscopy *in situ* (Fig. 2B and Movie S1†). TPPS assemblies emit their own fluorescence (Fig. S9†), while CMC was made visible for observation by labeling it with a fluorescein dye (Fig. S10†).

Three stages of CMC-induced TPPS assembly were identified (Fig. 2A and B): (1) rapid adsorption of TPPS onto the CMC aggregate surface. The adsorption reached near saturation within 1 minute after the addition of TPPS. (2) A slow nucleation period, with no significant growth observed at around 12 minutes, indicating the system was in the nucleation stage. (3) A rapid growth process, occurring between 15 and 24 minutes. Quantitative statistical analysis of the thickness of the TPPS layer on the CMC aggregate surface over time revealed a typical nucleation-growth process (Fig. 2C and S5†). During this period, a sea urchin-like assembly structure formed on the CMC surface, indicating radial growth of TPPS on the CMC.

During the growth period, besides the abundant TPPS assemblies attached onto CMC aggregates, numerous discrete TPPS nanowires appeared at 21 and 23 minutes (Fig. 2B). These nanowires predominantly clustered around the CMC aggregates, with fewer found in more distant regions. The fluorescence density of TPPS at various radii around the CMC aggregate was statistically analyzed (Fig. 2D). The results aligned well with Fick's law (Fig. 2D, S6, and ESI Section 3.2† for the Fick's diffusion model), indicating that these TPPS nanowires fragmented from the CMC surface and subsequently diffused into the solution. Furthermore, real-time imaging demonstrated that these fragments actively promoted TPPS growth (Fig. 2E, S6, and Movie S1†), confirming that the fragments act as seeds for nucleation and growth. Additionally, the increasing number of fragments, along with the progressively larger size of the central CMC-TPPS assemblies, suggests that the broken ends of these assemblies, attached to the CMC surface, are undergoing further catalytic cycles of re-growth and fragmentation. Moreover, this phenomenon was not limited to the surface: sea urchin-like TPPS aggregates in solution also exhibited longitudinal extension of TPPS assemblies on the CMC surface, along with numerous nearby TPPS fragments (Fig. 2F, Movies S2 and S3†). These observations further indicate that TPPS undergoes radial growth on the surface of CMC in solution, accompanied by a significant spontaneous fragmentation process.

**Fig. 3:**
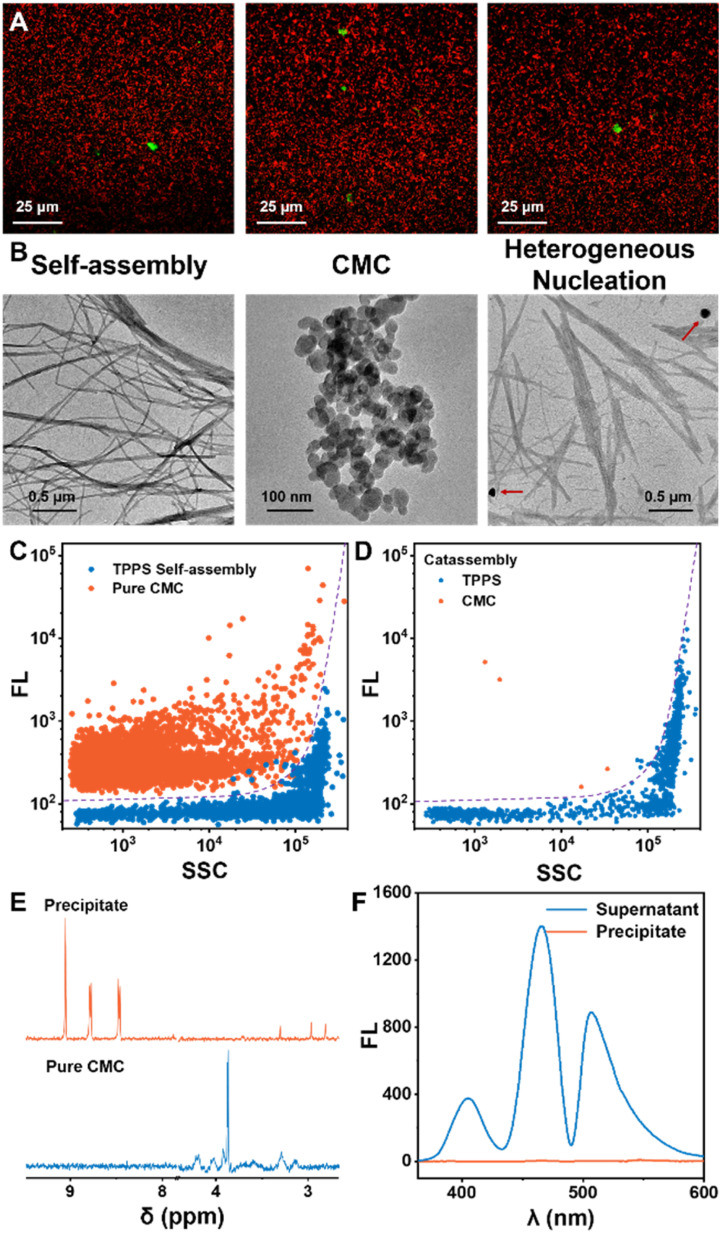
(A) CLSM overlay images illustrating the final state of the TPPS assembly solution with 0.5% equivalent CMC. Red dots: TPPS assemblies and green dots: CMC labeled by fluorescein. (B) TEM images of TPPS assemblies without addition of CMC, pure CMC, and TPPS assemblies with addition of 5% equivalent CMC. (C) Nano-flow cytometry analysis of pure CMC and TPPS assemblies, and a support vector machine (SVM) classification method was established. (D) Classification of the signals of TPPS assemblies induced by 5% equivalent CMC. (E) NMR spectra of the precipitate obtained by centrifuging a solution containing TPPS assemblies with 5% equivalent CMC, redissolved in D_2_O, compared with the NMR spectra of pure CMC. (F) Fluorescence spectra of the precipitate obtained by centrifuging a solution containing TPPS assemblies with 5% equivalent CMC–AHC, redissolved in H_2_O, compared with the fluorescence spectra of the supernatant. *E*_x_ = 344 nm.

The significant autocatalytic pathways enable chiral inducers to avoid interfering with the assembly and morphology of the final products, unlike classical template co-assembly methods. *In situ* confocal fluorescence imaging demonstrated the generation of abundant TPPS assemblies in the system, with only a small amount of CMC present, and a notable proportion of TPPS assemblies evidently not bound to CMC (Fig. 3A). TEM results revealed that the morphology of TPPS assemblies induced by CMC resembled nanowires formed by self-assembly, with a diameter of approximately 20 nm (Fig. 3B, S12 and S13†), which further aggregated into bundles. CMC showed a spherical morphology with a diameter of around 50 nm, with no binding observed between the two (Fig. 3B, S11 and S13†).

Moreover, nano-flow cytometry analysis of the final state of the assembly solution, based on fluorescence and scattering, clearly indicated that the predominant TPPS assemblies in the solution were not associated with CMC (Fig. 3C and D). Additionally, the assembly product was isolated by centrifugation, re-dissolved in water (or deuterated water), and characterized using NMR and fluorescence (Fig. 3E, F, S8 and S9†). NMR did not detect characteristic signals of CMC, and fluorescence spectroscopy also did not detect trace amounts of CMC in the TPPS assemblies, further demonstrating that in the final state of assembly, the vast majority of TPPS assemblies do not co-assemble with the nucleating agent CMC.

According to the above observations, it is proposed that the high efficiency of CMC in inducing TPPS supramolecular chiral polymerization can be attributed to two main factors. On the one hand, CMC itself, acting as a heterogeneous nucleating agent, was found to effectively promote TPPS primary nucleation, thereby achieving supramolecular chiral induction. On the other hand, the generated supramolecular chiral polymers undergo spontaneous fragmentation, which leads to two secondary pathways. First, the broken ends of these assemblies, attached to the CMC surface, undergo further catalytic cycles of re-growth and fragmentation. Second, this fragmentation-re-growth catalytic cycle generates a large number of chiral fragments, which then serve as seeds for nucleation and growth. Considering that the proportion of repeating units at the surface relative to the total repeating units of a single CMC particle is approximately 7%, the extent of chiral polymerization achieved on this surface is approximately 0.3% with the addition of a 0.5% equivalent of CMC (see ESI Section 3.5†). Therefore, the fragmentation-induced secondary pathway may make a significant contribution of around 99.7%, resulting in the rapid asymmetry amplification of supramolecular chirality.

### Structure–function relationship of chiral induction in heterogeneous nucleation

**Fig. 4:**
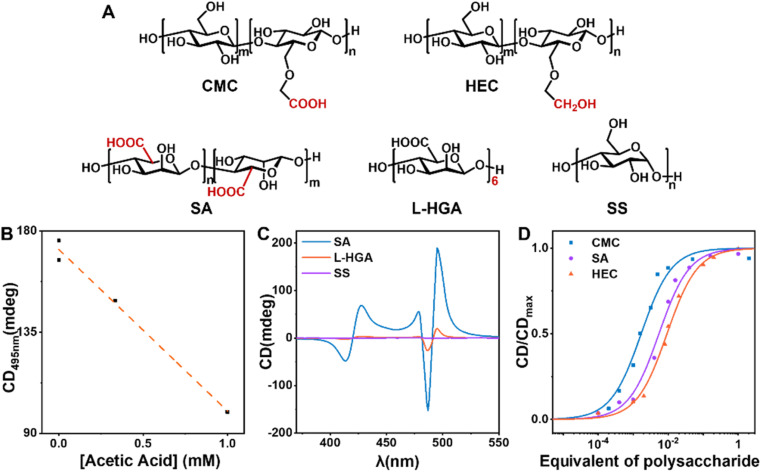
(A) Molecular structures of different nucleating agents. (B) The correlation between CD intensity at 495 nm of the final TPPS assemblies and acetic acid concentration. (C) CD spectra of TPPS assemblies induced by 10% equivalent SS, l-HGA, and SA. (D) CD intensity of TPPS assemblies *versus* equivalents of CMC, HEC, and SA, respectively.

To investigate the active sites and structural characteristics of CMC in efficient chiral induction, a systematic study of the structure–activity relationship was conducted. Using 10% equivalent of soluble starch (SS), which lacks side-chain modification and has a different main chain structure, as a control, the final TPPS assemblies exhibited almost no CD signal (Fig. 4C, S19 and S20†). This indicates that the side chains of CMC serve as key active sites for adsorbing monomers. Hydroxyethyl cellulose (HEC), differing from CMC only in the absence of carbonyl groups in the side chains, showed a critical saturated catalytic equivalent of 4%, much higher than 0.5% of CMC (Fig. 4D, S15 and S16†). This suggests a weaker binding between HEC and TPPS, necessitating a higher concentration to achieve the same induction effect, confirming that the carboxyl group is the key active site of the nucleating agent. To validate the hypothesis that CMC adsorbs TPPS monomers mainly through hydrogen bonding between the carboxyl group of CMC and the sulphonic group of TPPS, acetic acid was introduced as a hydrogen bonding competitor. Acetic acid was supposed to destroy the interactions between CMC and TPPS by forming hydrogen bonds with TPPS competitively, and thereby block the chiral transfer from CMC to TPPS assemblies. A significant reduction in CD intensity (Fig. 4B and S21†) of the TPPS assemblies upon addition of acetic acid was observed, confirming that CMC induces single enantiomer assembly mainly through hydrogen bonding.

However, sodium alginate (SA), a water-soluble polysaccharide with carboxyl groups, increased the critical saturated catalytic equivalent to 5% (Fig. 4D, S17 and S18†). This suggests that the flexible side chains in CMC may also play a key role. They may help retain appropriate degrees of freedom for the combined TPPS, allowing TPPS to adjust its conformation and spatial position, thereby facilitating the binding between TPPS towards nucleation.

Furthermore, l-hexaguluronic acid (l-HGA) was used to assess the influence of molecular weight on the efficiency of the nucleating agent. Despite the same equivalent, the induction effect of oligosaccharides was notably weaker than that of polysaccharides CMC (Fig. 4C, S19 and S20†). This underscores that heterogeneous nucleating agents require a certain degree of polymerization, aligning with our initial hypothesis that such agents require a specific surface area to sustain the stable formation of nuclei and initiate the primary nucleation process.

### The nucleating agent presents an optimal equivalent, and the heterogeneous nucleation follows the Eley–Rideal mechanism

**Fig. 5:**
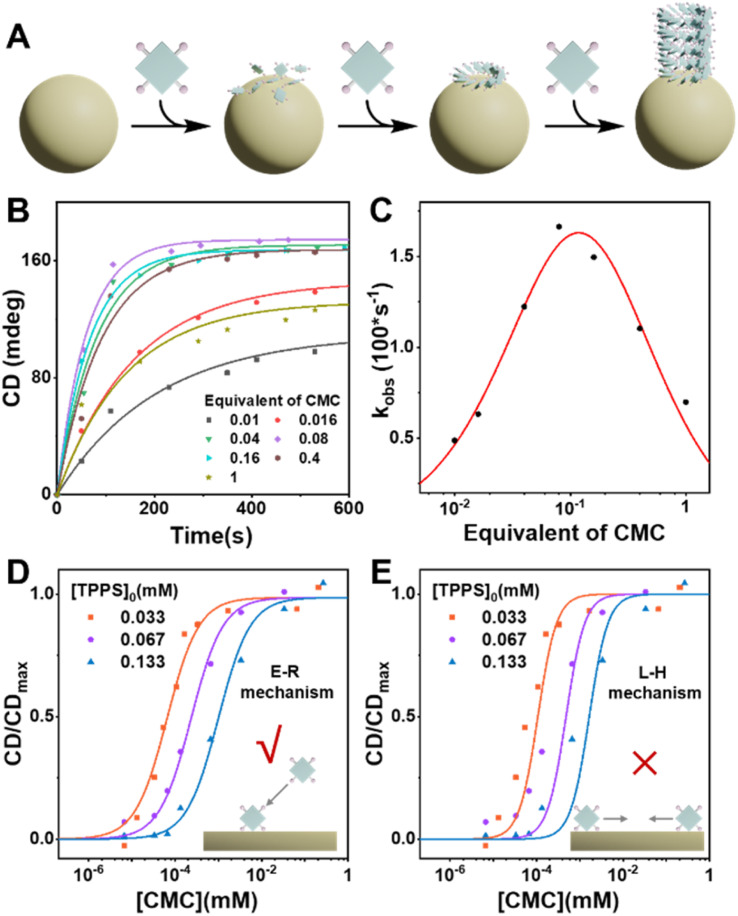
(A) Schematic representation of the CMC-induced heterogeneous nucleation-growth process of TPPS *via* the E–R mechanism. (B) CD kinetic curves of TPPS assembly with the addition of different equivalents of CMC, with the monomer concentration of TPPS set at 0.033 mM. (C) Apparent rate constant of TPPS assembly as a function of CMC equivalents, with fitting results using the E–R mechanism. (D) Fitting of CD/CD_max_–[CMC] curves at different TPPS concentrations using the E–R mechanism. (E) Fitting of CD/CD_max_–[CMC] curves at different TPPS concentrations using the L–H mechanism.

Kinetic results indicate that there is an optimal equivalent for heterogeneous nucleation, as the assembly rate of TPPS significantly decreased in the presence of high equivalents of the nucleating agent CMC (Fig. 5B, C, S26, and Table S5†). Excess nucleating agent inhibits the assembly process. To investigate the cause of this phenomenon, we compared two common surface catalytic mechanisms: the Langmuir–Hinshelwood (L–H) mechanism,^44^ where substrates adsorb and diffuse on the surface to form products, and the Eley–Rideal (E–R) mechanism,^45^ where the catalyst first adsorbs one substrate that reacts with another in solution. We hypothesize that this inhibitory trend arises from the decreasing concentration of free TPPS monomers in solution as CMC concentration increases, which slows the growth rate. This suggests that TPPS monomers adsorbed on the CMC surface assemble with those in solution, resembling the E–R mechanism of surface catalysis (Fig. 5A).

Based on this hypothesis, heterogeneous assembly kinetic models based on E–R mechanism were developed (see ESI Section 5.4†), and the relationship between the apparent rate constant and varying equivalents of CMC was fitted using this model. The results indicate a good fit (Fig. S27 and Table S6†), validating that CMC primarily promotes TPPS assembly through the E–R mechanism. Additionally, the curves of enantioselectivity (CD/CD_max_) against varying concentrations of CMC at different TPPS concentrations were fitted to kinetic models based on the E–R and L–H mechanisms (Fig. 5D, E, S24, and S25; see ESI Sections 5.2 and 5.3† for the kinetic models for E–R and L–H mechanisms, and original spectra in Fig. S2, S22 and S23†). The results demonstrate that the E–R mechanism model fits the curves well, while the L–H mechanism model performs poorly, further supporting our speculation regarding the E–R mechanism.

## Conclusions

This research demonstrates that CMC, as a heterogeneous nucleating agent, exhibits high efficiency in chiral induction, achieving maximum enantiomeric selectivity at low molar equivalents. This high efficiency in inducing TPPS supramolecular chiral polymerization can be attributed to two main factors. First, CMC itself promotes TPPS primary nucleation, facilitating supramolecular chiral induction. Second, the generated supramolecular chiral polymers undergo a catalytic cycle of spontaneous fragmentation and re-growth at their termini, with the resulting fragments also acting as seeds for nucleation and growth. Furthermore, exploring structure–function relationships reveals that key factors—such as the polymerization degree, flexibility of side chains, and the affinity between CMC and TPPS—significantly influence nucleation efficiency, aiding in the rational design of heterogeneous nucleating agents. Kinetic analysis reveals that an optimal equivalent exists in heterogeneous nucleation, following the E–R mechanism. In this process, TPPS monomers adsorbed on the CMC surface assemble with those in solution rather than through surface diffusion.

In summary, our study demonstrates that the surface enrichment-induced primary nucleation, along with substantial spontaneous fragmentation-induced autocatalysis, accounts for the high efficiency of the heterogeneous nucleation strategy. Unlike classical template co-assembly strategies, most assemblies remain unbound to the nucleating agent, allowing it to not interfere with the final assembly structure and morphology, akin to the role of a catalyst (or catassembler as proposed by Tian *et al.*^46,47^). This work advances the mechanistic understanding of heterogeneous nucleation and highlights its potential as an effective pathway control strategy in supramolecular polymerization systems.

## Data availability

The data supporting this article have been included as part of the ESI.†

## Author contributions

Peichen Shi: data curation, conceptualization, formal analysis, methodology and writing – original draft; Ganyu Chen: data curation, writing – original draft, and review & editing; Qiang Chen, Huiting Wu and Suixu Li: data curation and validation; Xiaoyu Cao, Liulin Yang and Zhongqun Tian: project administration and resources.

## Conflicts of interest

The authors declare no competing financial interest.

## Supplementary Material

SC-016-D4SC07894B-s001

SC-016-D4SC07894B-s002

SC-016-D4SC07894B-s003

SC-016-D4SC07894B-s004
