# Supplementary material for: Heterogeneously catalyzed supramolecular polymerization: essential roles of nucleation and fragmentation-induced autocatalysis in chiral transfer
Source: Chem Sci. 2025 Feb 18;16(13):5538–46. doi: 10.1039/d4sc07894b (PMC11862829; doi:10.1039/d4sc07894b)
Supplement: SC-016-D4SC07894B-s004 [file SC-016-D4SC07894B-s004.pdf]

Supplementary Information (SI) for Chemical Science.
This journal is © The Royal Society of Chemistry 2025

Supporting Information

Heterogeneously Catalyzed Supramolecular Polymerization: Essential Roles of Nucleation and Fragmentation-Induced Autocatalysis in Chiral Transfer

Peichen Shi,† Ganyu Chen,*,† Qiang Chen,† Huiting Wu,† Suixu Li,† Xiaoyu Cao,*,† Liulin Yang,*,† Zhongqun Tian*,†

†State Key Laboratory of Physical Chemistry of Solid Surface, Key Laboratory of Chemical Biology of Fujian Province, Collaborative Innovation Center of Chemistry for Energy Materials (iChEM), Innovation Laboratory for Sciences and Technologies of Energy Materials of Fujian Province (IKKEM), Department of Chemistry, College of Chemistry and Chemical Engineering, Xiamen University, Xiamen 361005 (P. R. China).

Table of Contents

1. Materials and Methods ··3

2. CMC accelerates nucleation and enhances enantioselectivity of TPPS supramolecular polymerization·····························5

3. The chiral transfer was amplified by autocatalytic fragmentation and growth ··7

3.1 CLSM image analysis of the thickness of TPPS layer.....................................7

3.2 Diffusion equation and fitting of the CLSM data 7

3.3 CLSM image analysis of the growth process of TPPS fragments 8

3.4 Characterization of TPPS assembly products with CMC as a nucleating agent 9

3.5 Estimation of the proportion of surface primary nucleation growth 11

4. Structure-function relationship of chiral induction in heterogeneous nucleating···14

5. Surface assembly mechanism ······································18

5.1 CD spectra of different concentration of TPPS with CMC 18

5.2 CD/CD_{max} – [CMC] model of E-R mechanism and global fitting of CD/CD_{max} – [CMC] curve 19

5.3 CD/CD_{max} – [CMC] model of L-H mechanism and global fitting of CD/CD_{max} – [CMC] curve 21

5.4 CD kinetic curves of 0.033 mM TPPS induced by different equivalents of CMC and fitting of k_{obs} – equivalent curve using E-R kinetic model.................22

1. Materials and Methods

Materials: TPPS, CMC, SA, SS, 3-azido-7-hydroxy-2h-chromen-2-one (AHC), and mono-Propargylamine were commercially available from Energy Chemical. 5(6)-Rhodamine Green amine was commercially available from DuoFluor Inc. HEC and L-HGA were commercially available from Shanghaiyuanye Bio-Technology.

Experimental conditions

A round-bottom flask was first filled with 1 mL of 1 M hydrochloric acid solution. A precision syringe pump (Harvard Pump) was then used to add 1 mL of TPPS solution at the desired concentration dropwise to the vortex edge of the stirring solution at a rate of 2 mL/min. The stirring speed was maintained at 940 rpm throughout the addition. Upon completing the addition, stirring continued for an additional 30 seconds before being stopped immediately, after which measurements were initiated. The reproducibility of this procedure is as follows:

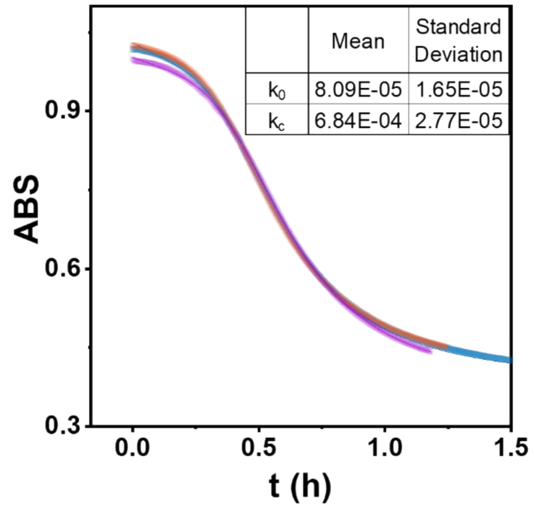

Figure S1. Three parallel sets of UV-Vis kinetic curves for the self-assembly process, with the monomer concentration of TPPS set at 3 μM.

Synthesis of CMC-AHC:

CMC (0.046 mM) was dissolved in 2 mL of MES buffer (pH 5.0) and stirred at 4 °C for 30 minutes. Subsequently, 21.17 mg of Sulfo-NHS and 19.17 mg of EDC were added, and the mixture was stirred for an additional 30 minutes before adjusting the pH to 7.6. Then, 20 mL of propargylamine was added to 10 mL of phosphate buffer (pH 7.5), and 0.5 mL of this propargylamine solution was incorporated into the first mixture, which was stirred for 24 hours. The resulting solution was dialyzed in a dialysis bag with a molecular weight cut-off of 3500 for 2 days and subsequently freeze-dried to obtain a pale-yellow powder.

The yellow precipitate was then dissolved in 5 mL of deionized water and 1 mL of DMSO, followed by the addition of 5 mL of a copper(I) chloride and ascorbic acid solution. This mixture was stirred at room temperature for 12 hours. Dialysis was performed using a dialysis bag with a molecular weight cut-off of 3500 until no fluorescence was observed in the dialysate, after which the solution was freeze-dried to yield another pale-yellow powder.

Synthesis of CMC-R6G:

CMC (0.046 mM) was dissolved in 2 mL of MES buffer (pH 5.0) and stirred at 4 °C for 30 minutes. Subsequently, 21.17 mg of Sulfo-NHS and 19.17 mg of EDC were added, and the mixture was stirred for an additional 30 minutes before adjusting the pH to 7.6. Then, 0.1 mg of 5(6)-Rhodamine Green amine was added to 10 mL of phosphate buffer (pH 7.5), and 0.5 mL of this propargylamine solution was incorporated into the mixture, which was stirred for 24 hours. The resulting solution was dialyzed in a dialysis bag with a molecular weight cut-off of 3500 for 2 days and then freeze-dried to yield a light-pink powder.

Methods:

Nuclear Magnetic Resonance (NMR) Spectroscopy: ^{1}H NMR was performed using a Bruker Avance III 600 MHz spectrometer equipped with a BBFO Cryoprobe.

Ultraviolet-visible (UV-vis) Spectroscopy: The UV-vis spectra were recorded using a Shimada UV-2700 spectrophotometer.

Transmission Electron Microscope (TEM): TEM imaging was conducted using a JEM-1400 microscope. A drop of the sample was first deposited onto a hydrophobic copper grid and allowed to rest for two minutes. The excess solution was then removed with a clean wiper. The sample was negatively stained with 2% uranyl acetate for 5 minutes and air-dried overnight. TEM images were acquired at an accelerating voltage of 100 kV.

Confocal Laser Scanning Microscopy (CLSM): CLSM imaging was performed using a Leica SP8 3X. A small amount of CMC-R6G and TPPS was placed onto the surface of a confocal dish, and imaging began immediately. The mixture was photographed at 3-second intervals over several minutes.

Fluorescence spectroscopy (FL): Fluorescence measurements were conducted using Hitachi F-7100 and F-4500 spectrometers.

Circular Dichroism Spectroscopy (CD): CD measurements were carried out using a Jasco J-810 spectrometer.

Nano-Flow Cytometry (n-FCM): n-FCM was performed using a custom-built nano-flow cytometer.[1]

2. CMC accelerates nucleation and enhances enantioselectivity of TPPS supramolecular polymerization

The self-similar autocatalytic model of TPPS nucleation and growth process follow the empirical formula[2, 3]:

$$Abs = A * \left(1 + \frac{(m-1) * (k_0 * x + (k_c * x)^{n+1})}{n+1}\right)^{\frac{-1}{m-1}} + B$$

Here, $\boldsymbol{k_0}$ represents the rate constant for self-assembly, $\boldsymbol{k_c}$ represents the rate constant for autocatalysis, ***m*** represents the number of nuclei, and ***n*** represents the reaction order of the autocatalytic process. ***A*** and ***B*** are normalization constants.

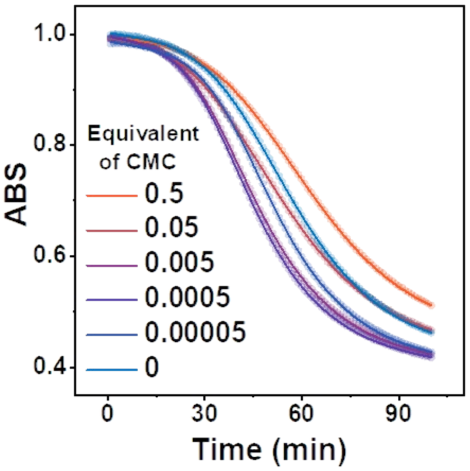

Figure S2. Kinetic curves of 3 μM TPPS assembly under varying equivalents of CMC. Spectra were obtained by monitoring absorbance at 434 nm and fitted using the aforementioned nucleation and growth model.

Table S1. Nucleation and growth model fitting results for UV kinetic curves of 3 μM TPPS assembly

**e.q.**	***A***	***B***	$\boldsymbol{k_0}$**(M^{-1}s^{-1})**	$\boldsymbol{k_c}$**(s^{-1})**	***m***	***n***	$\boldsymbol{R^2}$
0	0.591	0.411	0.00165	0.0240	1.85	2.93	1.00
5E-5	0.599	0.388	0.00177	0.0269	2.13	3.41	1.00
5E-4	0.610	0.387	0.00229	0.0310	2.13	3.03	1.00
0.005	0.611	0.388	0.00222	0.0301	2.10	2.91	1.00

0.05	0.578	0.416	0.00171	0.0251	1.69	2.23	1.00
0.5	0.545	0.450	0.00120	0.0214	1.52	2.42	1.00

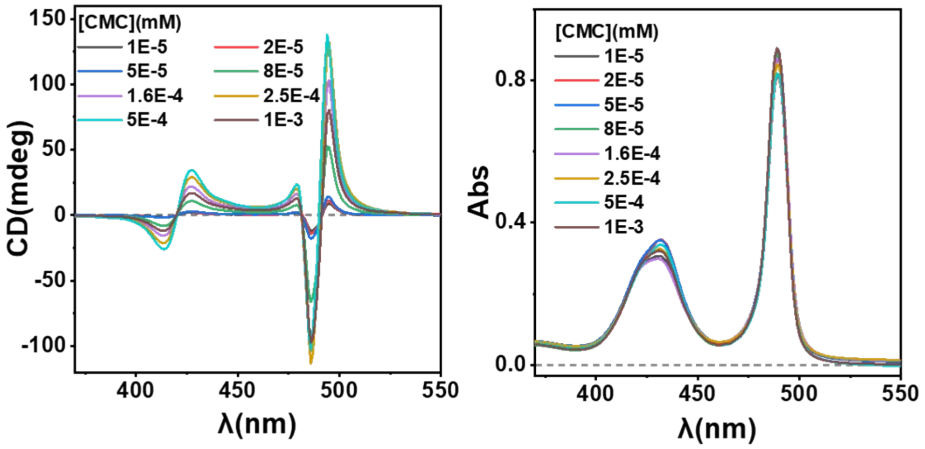

Figure S3. CD and UV-Vis spectra of 0.033 mM TPPS with varying equivalents of CMC incorporated.

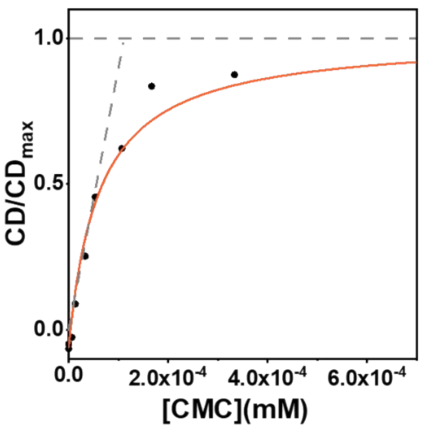

Figure S4. The CD/CDmax values at 495 nm for TPPS assemblies versus different concentration of CMC. The gray dashed line represents the initial slope and a conversion rate of 1.

Identifying the critical saturated equivalent for achieving maximum enantioselectivity from the CD/CD_{max} - [CMC] curve proved challenging. Consequently, we determined the equivalent as the amount of nucleating agent required for 50% conversion, at which the rates of self-assembly and heterogeneous nucleation are equal. We then used twice of this equivalent to determine the critical saturated

equivalent. At a TPPS concentration of 0.033 mM, the critical saturated equivalent was found to be 0.5%.

3. The chiral transfer was amplified by autocatalytic fragmentation and growth

3.1 CLSM image analysis of the thickness of TPPS layer

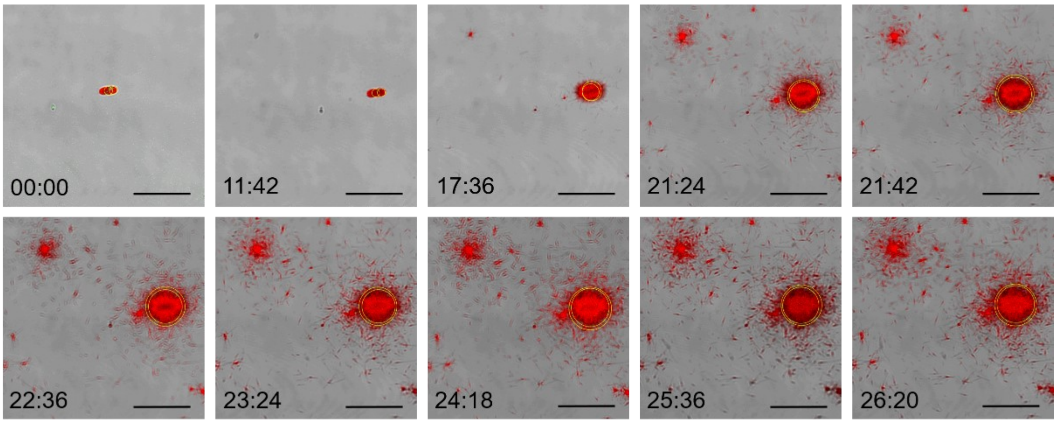

Figure S5. Real-time CLSM overlay images illustrating the adsorption of TPPS on the surface of CMC and the subsequent radial growth of TPPS polymers. Yellow circles indicate regions used for statistical analysis of the thickness of CMC and TPPS aggregates. Note: Scale bar 10 μm.

3.2 Diffusion equation and fitting of the CLSM data

If the TPPS nanowires around CMC are primarily formed by fragments originating from the CMC surface and subsequently diffusing, their distribution in relation to the distance from the center should follow Fick's second law:

$$\frac{\partial c(R,t)}{\partial t} = - D\nabla c(R,t)^2$$

Furthermore, we assume initial conditions as $c(0,0) = \delta c$, then we could get:

$$c(R,t_0) = \frac{c_0}{\sqrt{4\pi D t_0}} e^{-\frac{R^2}{4Dt_0}}$$

Here, ***R***, ***t*** and ***D*** represent the distance from the CMC center, time, and diffusion coefficient, respectively. ***c*(*R*,*t*)** denotes the concentration of TPPS assemblies in a concentric ring at distance ***R*** and time ***t***.

Assuming that the fluorescence intensity is linearly correlated with concentration at the current concentration, the fitting equation can be expressed as follows:

$$I(r,t_0) = \frac{A}{\sqrt{4\pi D t_0}} e^{-\frac{r^2}{4Dt_0}} + B$$

A and ***B*** represent the normalization constants.

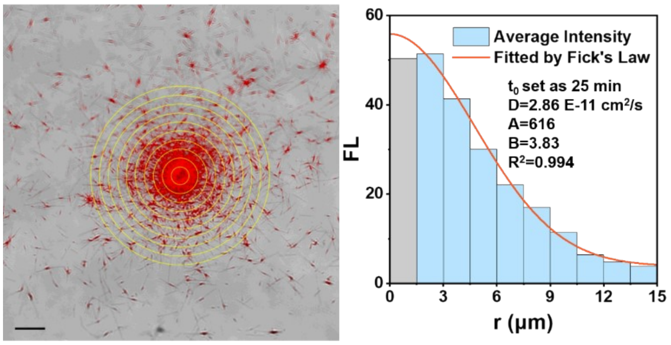

Figure S6. Analysis of fluorescence signal values between adjacent concentric circles at different distances from the center, and fitting results according to Fick's law. Note: Scale bar 5 μm.

3.3 CLSM image analysis of the growth process of TPPS fragments

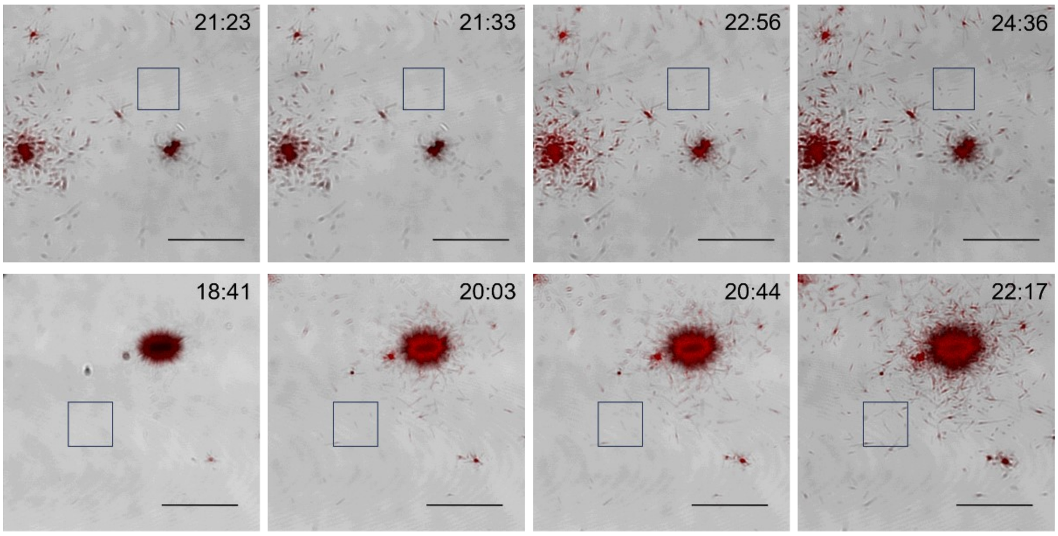

Figure S7. CLSM overlay images at various time points showing the growth process of TPPS fragments diffusing from the CMC particle surface. For ease of observation, the nanowire fragments exhibiting this phenomenon are highlighted with dark-blue frames. Note: Scale bar 10 μm.

3.4 Characterization of TPPS assembly products with CMC as a nucleating agent

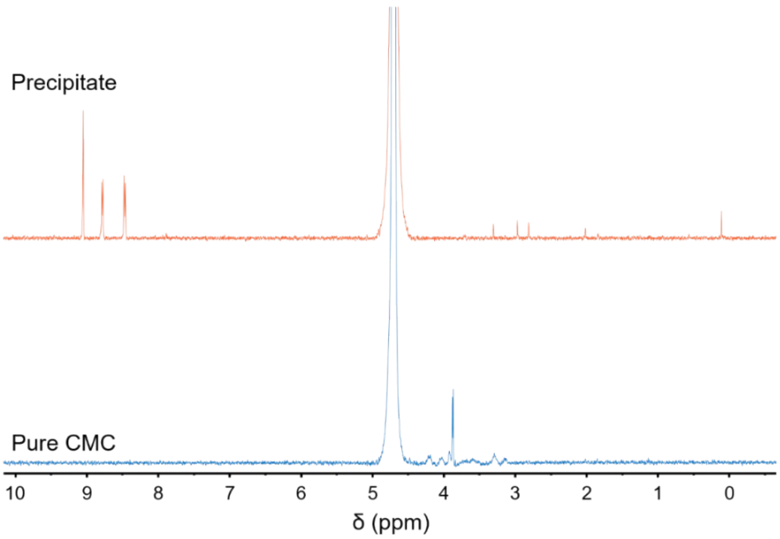

Figure S8. NMR spectrum of the assembly product precipitate from the system containing 0.033 mM TPPS and 5% equivalent CMC, redissolved in D_2O, alongside the spectrum of pure CMC.

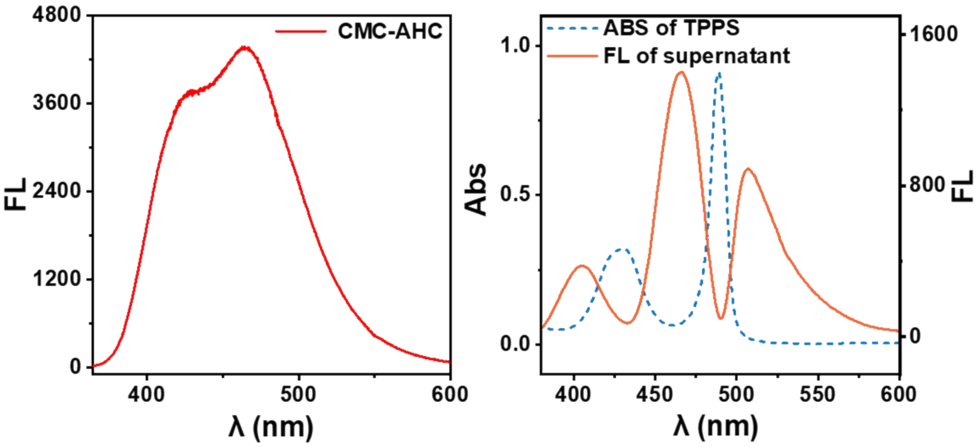

Figure S9. FL emission spectroscopy (λ_{ex} = 344 nm) of pure CMC-AHC and the supernatant from the system containing 0.033 mM TPPS and 5% equivalent CMC-AHC.

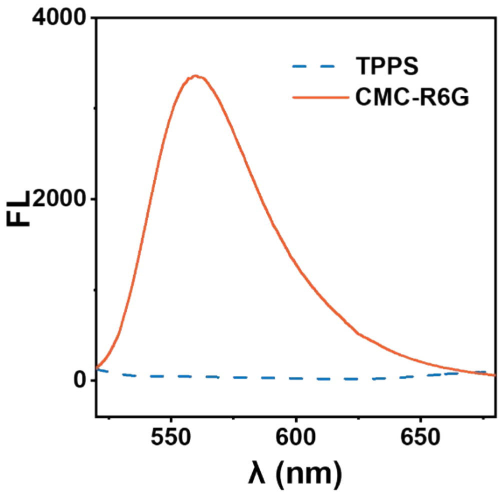

Figure S10. FL emission spectroscopy (λ_{ex} = 488 nm) of pure CMC-R6G and TPPS assemblies.

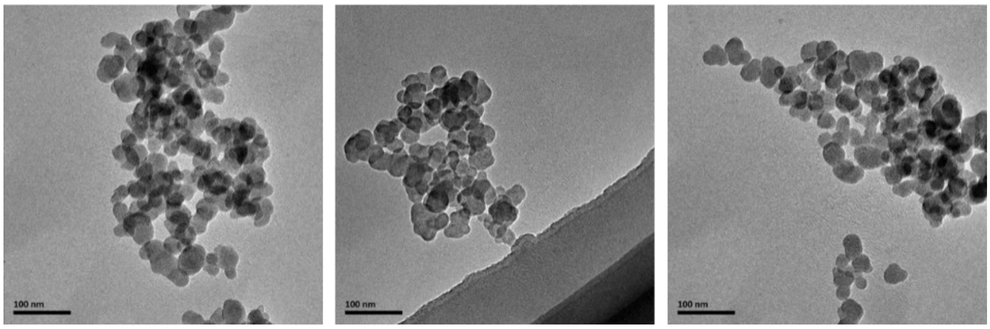

Figure S11. TEM images of the dried solid from a 5 μM CMC solution.

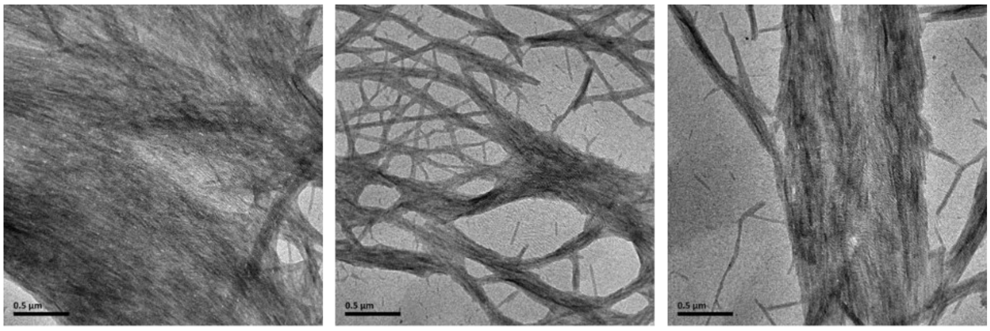

Figure S12. TEM images of the dried solid from a 0.033 mM TPPS assembly solution.

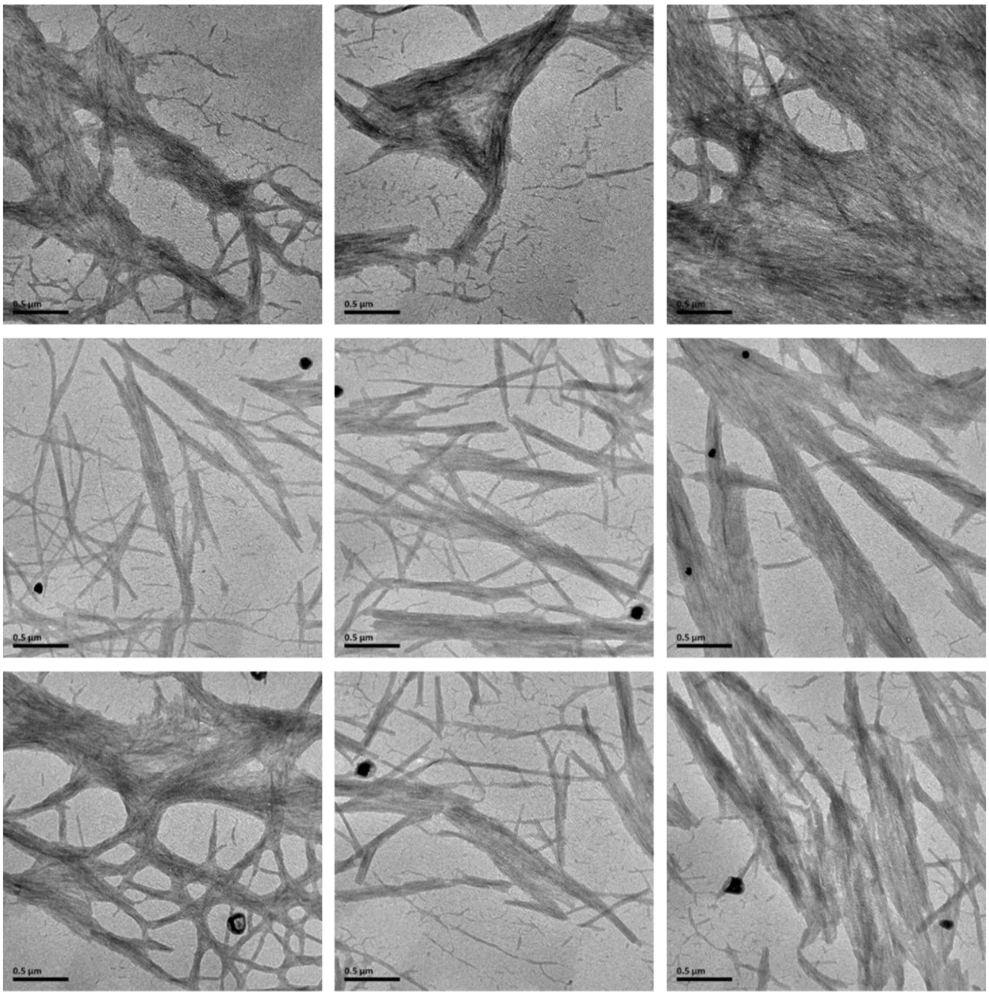

Figure S13. TEM images of the dried solid from a 0.033 mM TPPS assembly solution with the addition of 5% equivalent CMC as a nucleating agent.

3.5 Estimation of the proportion of surface primary nucleation growth

Table S2. Parameters estimation used for calculating the surface assembly proportion

**Parameters**	**Estimation**
Diagonal length of TPPS monomer (***l***)	2 nm
Inclination Angle of TPPS in assemblies (ϑ)	30°
Width of a single TPPS nanowire ($\boldsymbol{L_n}$)	20 nm
Repeated unit size of CMC ***a***×***b***×***c***	5.58 Å×4.77 Å×3.07 Å (without side chain)<br>5.58 Å×7.63 Å×3.07 Å (with side chain)
Particle size of CMC in solid (***r***)	~ 25 nm

Length of TPPS assemblies on CMC (***L***)	~ 4 μm

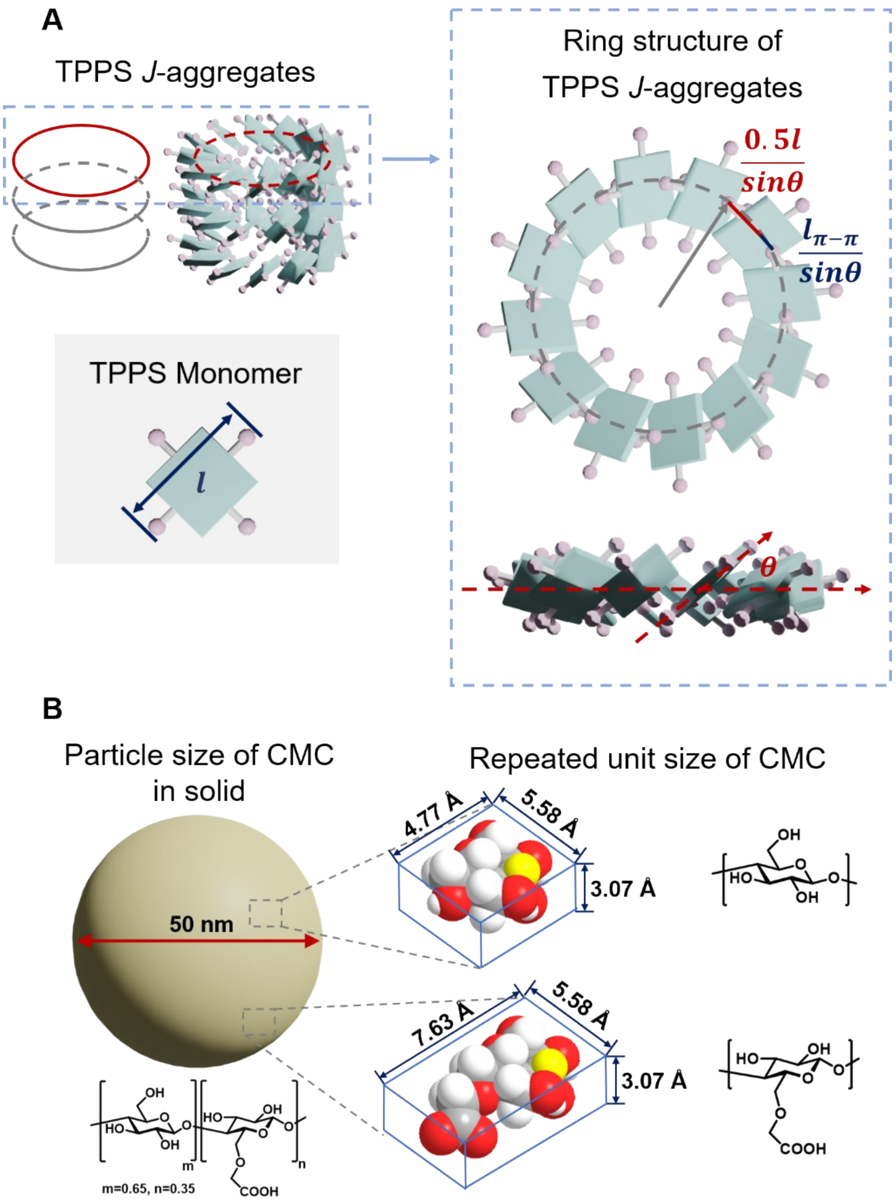

Figure S14. Schematic diagrams of minimal repeating unit structure of TPPS *J*-aggregates (A) and illustration of CMC and the estimation of repeating unit dimensions (B).

J-aggregates of TPPS are generally considered to form nanotube structures driven by electrostatic interactions between the sulfonate groups and protonated porphyrin moieties. In transmission electron microscopy (TEM) images, we observed that the diameter (L_n) of a single nanotube is approximately 20 nm. Due to the loss of solvent

protection, these nanotubes tend to adopt a flattened structure, and their circumference can be estimated as $2L_n$.

For the minimal structural unit of a TPPS nanotube, the size occupied by a repeating monomer can be estimated based on the molecular dimensions ($0.5l/sin\theta$) and the intermolecular spacing (π-π stacking distance, $0.3/sin\theta$) (Figure S14A). Therefore, the number of molecules in a ring-like structure[4] can be calculated using the following equation:

$$N_{nu} = \frac{2L_n}{0.5l + 0.3} sin\vartheta - 2 = 19.8$$

This is consistent with the minimum size of *J*-aggregates reported before[5-8] and further supports the validity of the parameter estimation regarding the inclination angle of TPPS in the assemblies.

The average degree of polymerization of CMC particles can be estimated using the following equation (***m***, ***n*** represent to the ratio of the two repeated unit in CMC):

$$DP_n = \frac{V_{CMC}}{V_M} = \frac{\frac{4}{3}\pi r^3}{(m + 1.6n)abc} = 6.71 \times 10^5$$

Here, DP_n represents the average number of repeating units contained in a single CMC particle, while V_{CMC} and V_M denote the volumes of the CMC particles and the CMC repeating units, respectively.

The average number of repeating units at the surface of a single CMC particle ($\boldsymbol{N_s}$) can be estimated as follows:

$$N_s = \frac{S_{CMC}}{S_M} = \frac{4\pi r^2}{ac} = 1.83 \times 10^5$$

Here, S_{CMC} and S_M denote the volumes of the CMC particles and the CMC repeating units, respectively.

The proportion of repeating units at the surface relative to the total repeating units of a single CMC particle (α) is as follows:

$$\alpha = \frac{N_s}{N_V} = 0.0682$$

The number of TPPS *J*-aggregates that can be adsorbed on a single CMC particle surface (η) is as follows:

$$\eta = \frac{S_{CMC}}{S_{nu}} = \frac{4\pi r^2}{\frac{1}{4}\pi L_n^2} = 25.0$$

S_{nu} represents the surface area of the ring structure of the TPPS *J*-aggregates.

The number of ring structures in the TPPS *J*-aggregates adsorbed on the CMC surface (N_p) can be estimated as follows:

$$N_p = \frac{L}{lsin\vartheta} = 4.00 \times 10^3$$

The proportion of TPPS remaining adsorbed on the CMC surface after assembly relative to the total TPPS concentration (γ) can be estimated as follows **(*eq* = 0.5%)**:

$$\gamma = \frac{\eta N_p eq}{DP_n} = 0.278\%$$

4. Structure-function relationship of chiral induction in heterogeneous nucleating

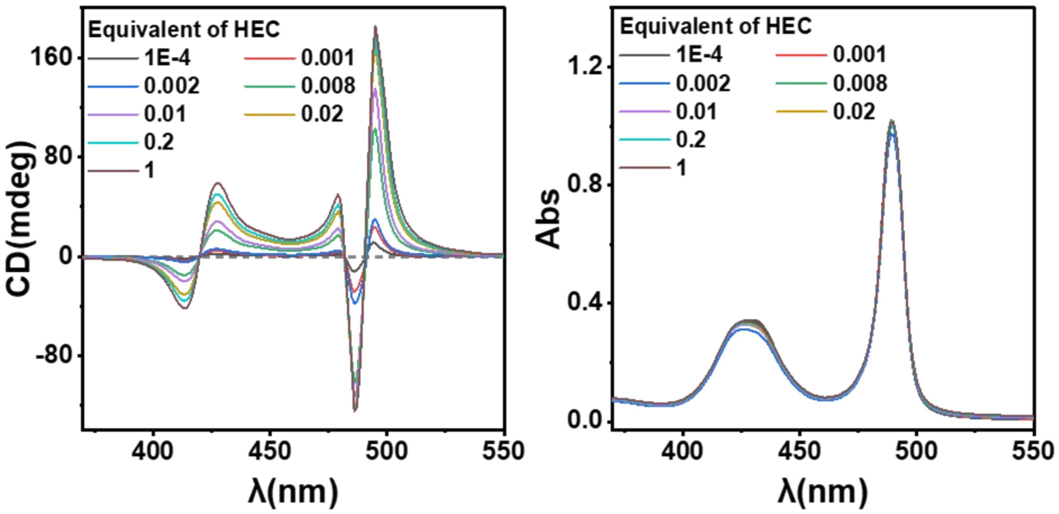

Figure S15. CD and UV-Vis spectra of 0.033 mM TPPS with varying equivalents of HEC incorporated.

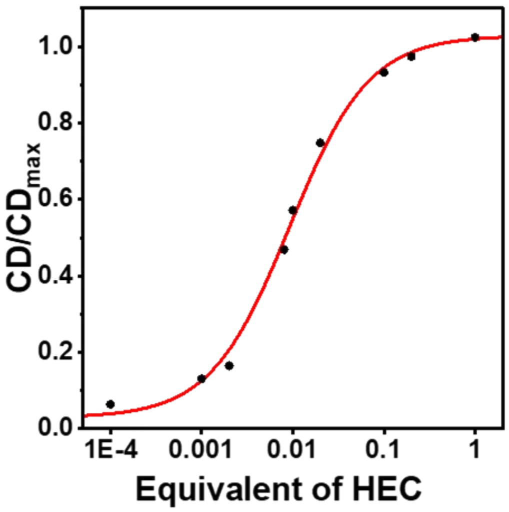

Figure S16. CD/CD_{max} - HEC equivalents curve and fitted with the E-R mechanism.

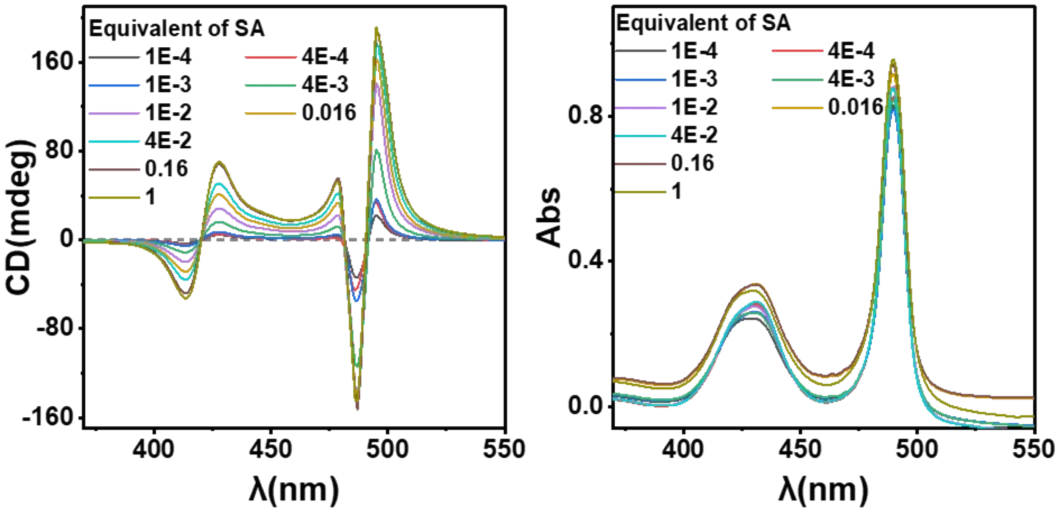

Figure S17. CD and UV-Vis spectra of 0.033 mM TPPS with varying equivalents of SA incorporated.

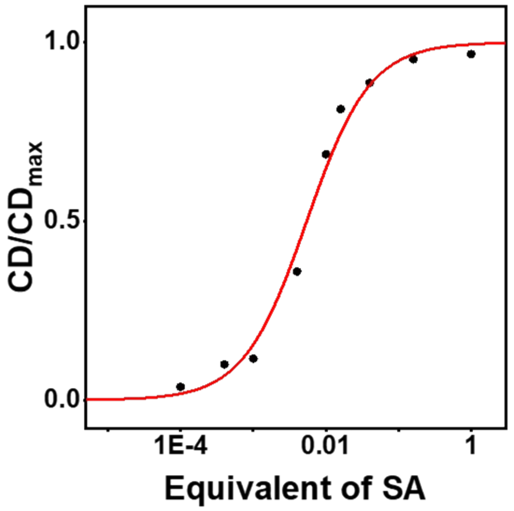

Figure S18. CD/CD_{max} - SA equivalents curve and fitted with the E-R mechanism.

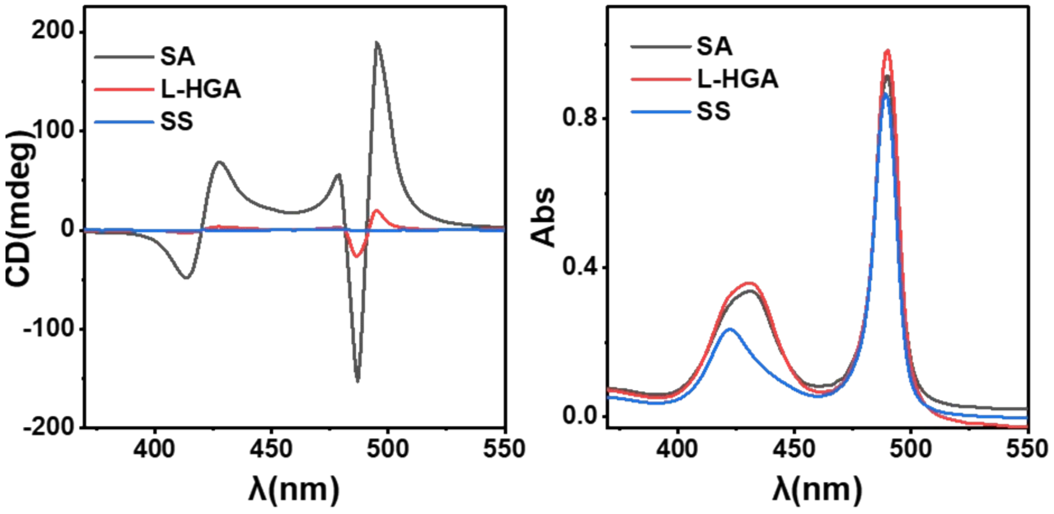

Figure S19. CD and UV-Vis spectra of 0.033 mM TPPS with the incorporation of 10% equivalents of SA, L-HGA, and SS.

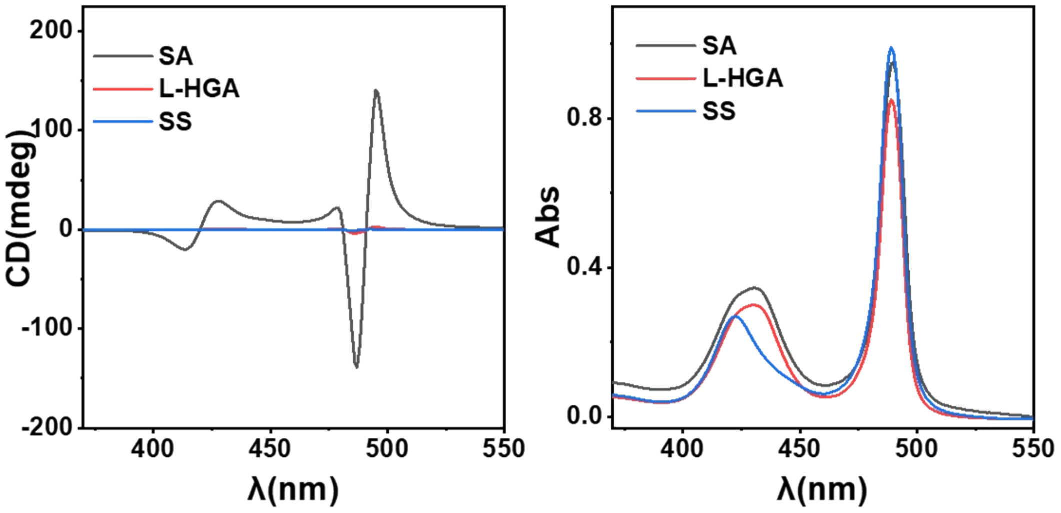

Figure S20. CD and UV-Vis spectra of 0.033 mM TPPS with the incorporation of 1% equivalent of SA, L-HGA and SS.

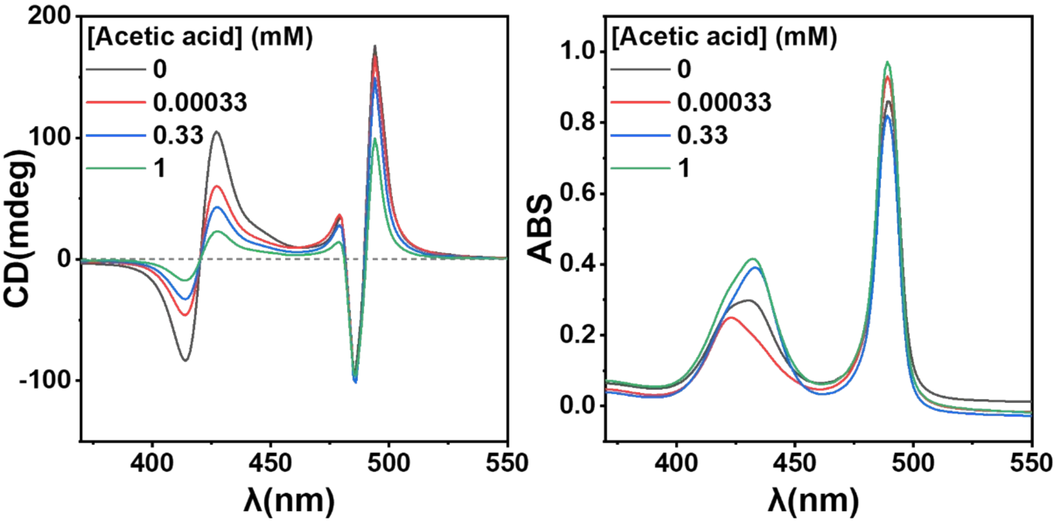

Figure S21. CD and UV-Vis spectra of 0.033 mM TPPS with 0.5% equivalent CMC under varying concentrations of acetic acid.

5. Surface assembly mechanism

5.1 CD spectra of different concentration of TPPS with CMC

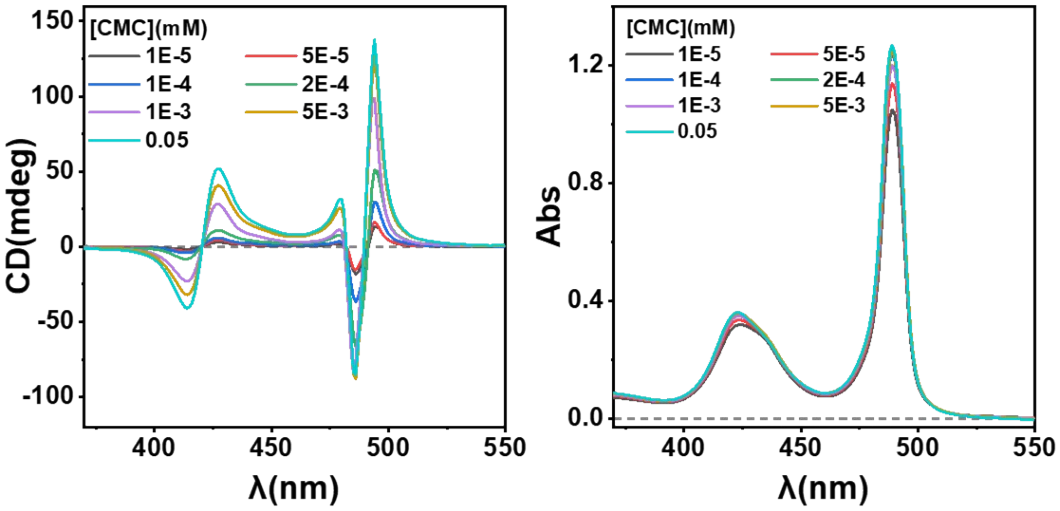

Figure S22. CD and UV-Vis spectra of 0.067 mM TPPS with varying equivalents of CMC incorporated.

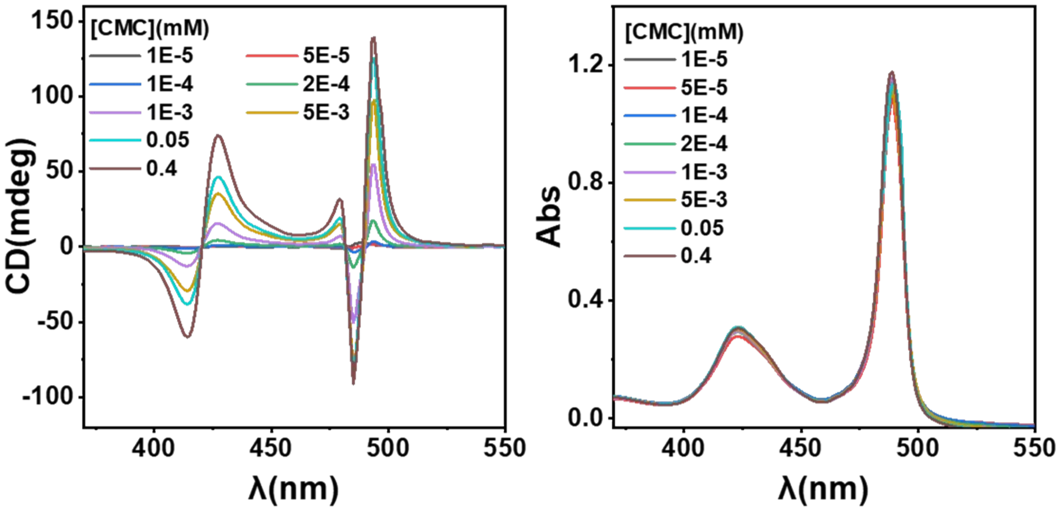

Figure S23. CD and UV-Vis spectra of 0.133 mM TPPS with varying equivalents of CMC incorporated.

5.2 CD/CD$_{max}$ – [CMC] model of E-R mechanism and global fitting of CD/CD$_{max}$ – [CMC] curve

The concentration of TPPS adsorbed on CMC surface (***θ***) was calculated as follows:

$$[CMC]_{free} + \theta = [CMC]_{free} + K[CMC]_{free}[TPPS] = [CMC]_0$$

$$[CMC]_{free} = \frac{[CMC]_0}{1 + K[TPPS]}$$

$$\theta = \frac{K[TPPS][CMC]_0}{1 + K[TPPS]}$$

Here, **[CMC]$_{free}$**, ***θ***, **[CMC]$_0$** represent the concentrations of free CMC, adsorbed CMC, and total CMC, respectively, while ***K*** denotes the binding constant between CMC and free TPPS.

The E-R mechanism describes the participation of TPPS adsorbed on the CMC surface and free TPPS in the nucleation process. The rate equation for this process is as follows:

$$r_{cat} = k_{ER}\theta^m[TPPS]^{n-m} = k_{ER}\frac{K^m{[CMC]_0}^m[TPPS]^n}{(1 + K\cdot[TPPS])^m}$$

Here, ***m*** represents the reaction order of molecules participating at the surface, while ***n*** denotes the overall reaction order of the catalytic process, with $m \geq 1$and $n > m+1$.

Because of the enantiomeric excess in the final assemblies, the assembly process is under kinetic control, with the ratio of rate constants dictating the ultimate enantiomeric selectivity:

$$e.e. = \frac{r_{cat}}{r_{cat} + r_{self}} = \frac{k_{ER}\frac{K^m{[CMC]_0}^m[TPPS]^n}{(1 + K\cdot[TPPS])^m}}{k_{ER}\frac{K^m{[CMC]_0}^m[TPPS]^n}{(1 + K\cdot[TPPS])^m} + k_{self}[TPPS]^p} = \frac{[illegible]}{k_{ER}K^m{[CMC]_0}^m + [illegible]}$$

Here, ***p*** is the reaction order of the self-assembly process, with $p \geq 2$.

Considering the chirality of the assemblies is determined by the chirality at the nucleation stage,[9] it is assumed that the initial enantiomeric selectivity dictates the final enantiomeric selectivity. Therefore, the equation can be simplified as follows:

$$e.e. = \frac{k_{ER}K^m[CMC]_0{}^m}{k_{ER}K^m[CMC]_0{}^m + k_{self}[TPPS]_0{}^{p-n}(1 + K\cdot[TPPS])^m} = \frac{[illegible]}{k_{ER}K^m\, eq^m + k_{self}\,[illegible]}$$

Here, $\boldsymbol{k_{ER}}$ and $\boldsymbol{K}$ represent the surface-catalyzed rate constants under the E-R mechanism and the binding constant of TPPS to CMC, respectively. $\mathbf{[TPPS]_0}$ and $\mathbf{[CMC]_0}$ denote the initial concentrations of TPPS and CMC. $\boldsymbol{m}$ and $\boldsymbol{n}$ refer to the number of adsorbed TPPS units and the total reaction order of the nucleation process, respectively. $\boldsymbol{p}$ denotes the reaction order of the nucleation process during self-assembly, while *eq* represents the equivalents of CMC (calculated by the repeating unit).

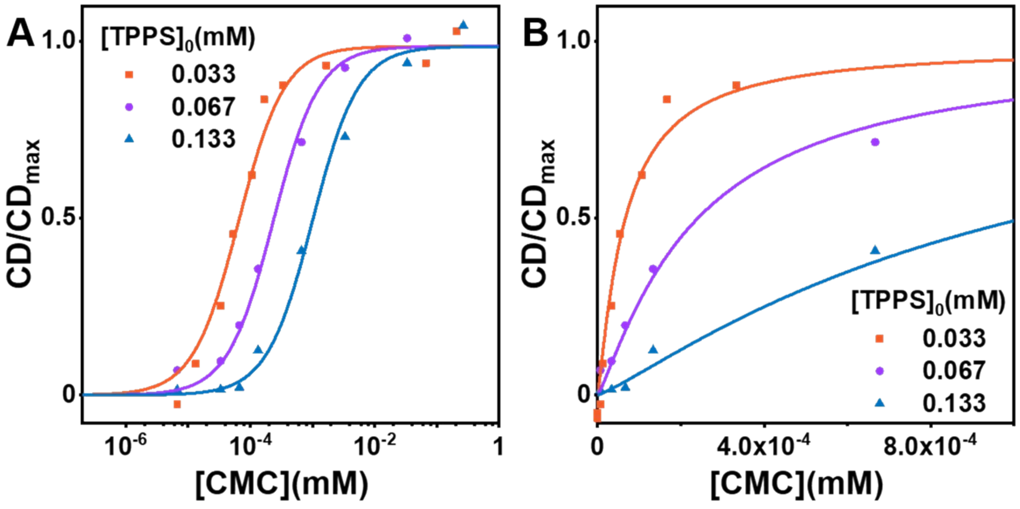

Figure S24. Global fit of CD/CD$_{max}$ - [CMC] in (A) logarithmic scale and (B) linear scale, using the E-R mechanism model.

Table S3. Parameters of the global fit of CD/CD$_{max}$ - [CMC] using E-R mechanism

**[TPPS]$_0$ (mM)**	$\boldsymbol{k_{ER}/k_{self}}$	$\boldsymbol{K}$ **(mM^{-1})**	$\boldsymbol{k_{self}}$ **(mM^{-1}s^{-1})**	$\boldsymbol{m}$	$\boldsymbol{p\text{-}n}$	$\boldsymbol{R^2}$
Set as 0.0333<br>Set as 0.0667<br>Set as 0.133	20.4 (global)	35.9 (global)	Set as 1	1.17 (global)	1.46 (global)	0.985 (global)

5.3 CD/CD$_{max}$ – [CMC] model of L-H mechanism and global fitting of CD/CD$_{max}$ – [CMC] curve

The Langmuir-Hinshelwood (L-H) mechanism involves the adsorption of two or more substrate molecules onto the surface, followed by diffusion and subsequent assembly. The rate equation for this process can be expressed as follows:

$$r_{cat} = k_{LH}\theta^m = \frac{k_{LH}\left(K[CMC]_0[TPPS]\right)^m}{(1 + K[TPPS])^2}$$

$$e.e. = \frac{r_{cat}}{r_{cat} + r_{self}} = \frac{\dfrac{k_{LH}\left(K[CMC]_0[TPPS]\right)^m}{(1 + K[TPPS])^m}}{\dfrac{k_{LH}\left(K[CMC]_0[TPPS]\right)^m}{(1 + K[TPPS])^m} + k_{self}[TPPS]^p}$$

$$= \frac{k_{LH}K^2[CMC]_0{}^m}{k_{LH}K^m[CMC]_0{}^m + k_{self}[TPPS]^{p-m}(1 + K[TPPS])^m}$$

Here, ***m*** represents the reaction order of the surface-catalyzed process, while ***p*** denotes the reaction order of the self-assembly process in solution. It is important to note that $m \geq 2$ in this context.

This equation can be simplified in a manner similar to the aforementioned E-R mechanism:

$$e.e. = \frac{k_{LH}K^2[CMC]_0{}^m}{k_{LH}K^m[CMC]_0{}^m + k_{self}[TPPS]_0{}^{p-m}(1 + K[TPPS]_0)^m}$$

Here, $\boldsymbol{k_{LH}}$ and $\boldsymbol{K}$ represent the surface catalyzed rate constants under L-H mechanism and the binding constant of TPPS and CMC. **[TPPS]$_0$** and **[CMC]$_0$** denote the initial concentrations.

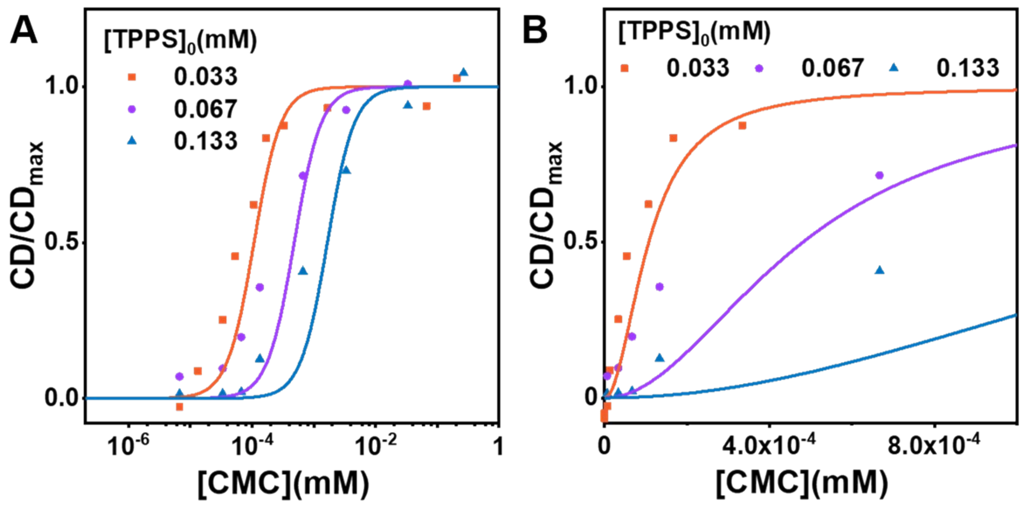

Figure S25. Global fit of CD/CD_{max} - [CMC] in (A) logarithmic scale and (B) linear scale using the L-H mechanism.

Table S4. Parameters from the global fit of CD/CD_{max} - $[CMC]_0$ using L-H mechanism

$[TPPS]_0$ (mM)	k_{LH}/k_{self}	K (mM^{-1})	k_{self} ($mM^{-1}s^{-1}$)	m	p	R^2
Set as 0.0333	260 (global)	0.976 (global)	Set as 1	2.00 (global)	5.63 (global)	0.897 (global)
Set as 0.0667						
Set as 0.133						

5.4 CD kinetic curves of 0.033 mM TPPS induced by different equivalents of CMC and fitting of k_{obs} – equivalent curve using E-R kinetic model

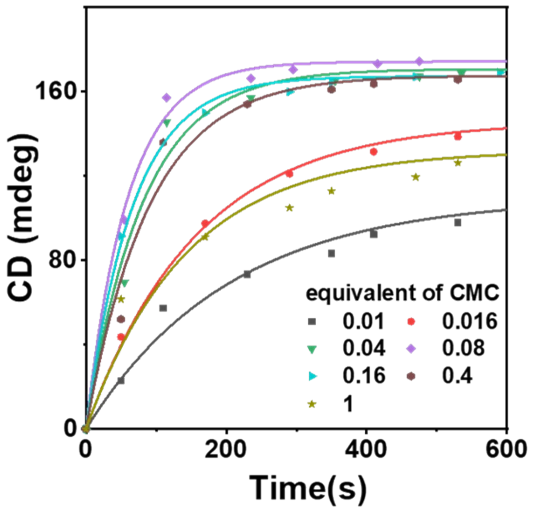

Figure S26. CD kinetic curves of different $[CMC]_0$ and fitting with first order kinetic model $y=(A-A*\exp(-k*x))$.

Table S5. First order kinetic fitting of CD kinetic curves

**e.q.**	***A*(mdeg)**	***k*(s^{-1})**	R^2
0.01	110	0.00487	0.975
0.016	146	0.00633	0.996
0.04	171	0.0123	0.976
0.08	174	0.0167	0.993
0.16	167	0.0150	0.995
0.4	168	0.0110	0.969
1	132	0.00699	0.922

The law governing the conservation of TPPS matter is as follows:

$$[TPPS]_{fc} + [TPPS]_f = [TPPS]_m$$

Here, $\mathbf{[TPPS]_{fc}}$ represents the total concentration of TPPS on CMC surface. $\mathbf{[TPPS]_f}$ represents the total concentration of TPPS in solution.

$$K'[CMC]_0[TPPS]_f + [TPPS]_f = [TPPS]_m$$

$$[TPPS]_f = \frac{[TPPS]_m}{K'[CMC]_0 + 1}$$

In the global fitting results of CD/CD_{max} - $[CMC]_0$ based on the E-R mechanism, the reaction order for surface-adsorbed TPPS (***m***) is approximately 1, with a difference of 1.46 between the overall reaction orders of self-assembly and heterogeneous nucleation (***p***-***n***). For simplification, we approximate this difference as 1 to facilitate calculations, leading to the following rate equation:

$$r_{cat} = k_{ER}\frac{K[CMC]_0[TPPS]_f^2}{1 + K[TPPS]_f} = \frac{kK[CMC]_0[TPPS]_m^2}{(K'[CMC]_0 + 1)^2 + K(K'[CMC]_0 + 1)[TPPS]_m}$$

The initial concentration of TPPS can be estimated using the following equation:

$$[TPPS]_m \approx [TPPS]_0 - [CMC]_0$$

$$r_{cat} = k_{ER}\theta[TPPS]_f = k_{ER}\frac{K[CMC]_0{[TPPS]^2}_f}{1 + K[TPPS]_f} = \frac{kK[CMC]_0[TPPS]_m^2}{(K'[CMC]_0 + 1)^2 + K(K'[CMC]_0 + 1)[TPPS]_m}$$

The observed kinetic curves follow a first-order kinetic process:

$$r_{cat} = k_{obs}[TPPS]_m$$

The relationship between the apparent rate constant and the equivalent concentration is as follows:

$$k_{obs} = \frac{kK[CMC]_0[TPPS]_m}{(K'[CMC]_0 + 1)^2 + K(K'[CMC]_0 + 1)[TPPS]_m} = \frac{kK[CMC]_0([\text{[illegible]}}{(K'[CMC]_0 + 1)^2 + K(K'[\text{[illegible]}} = \frac{kKeq(1 - eq)}{(K'eq + 1)^2 + K(K'eq + 1)(1 - eq)}$$

Here, ***k***, ***K***, and ***K'*** represent the rate constant for surface catalysis according to the E-R mechanism, the binding constant between CMC and TPPS monomers, and the average binding constant between CMC and both TPPS monomers and assemblies, respectively. And ***eq*** represents the molar equivalent of CMC.

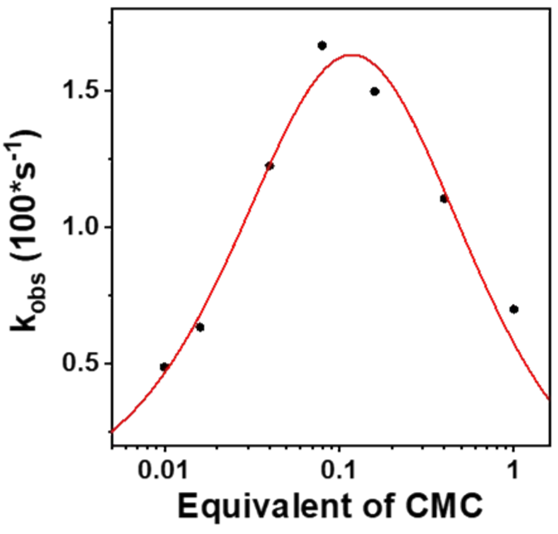

Figure S27. k_{obs} – equivalent curve and fitting using E-R kinetic model.

Table S6. Parameters from fitting of k_{obs} - equivalent curve using E-R kinetic model

C_0 (mM)	k_{ER} (s^{-1})	K (mM^{-1})	K' (mM^{-1})	R^2
Set as 0.033	8.44	65.5	4.42E3	0.956

5.5 Mechanisms of self-assembly and heterogeneous nucleator-mediated assembly process

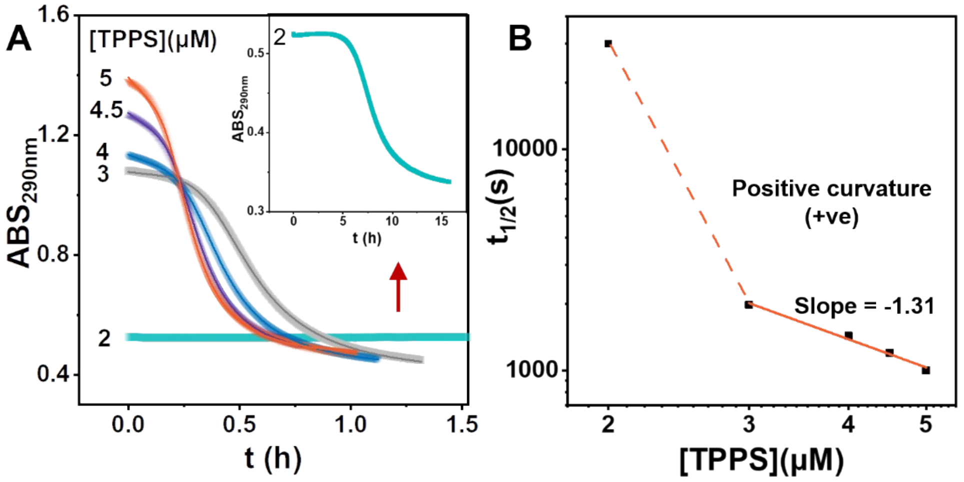

Figure S28. (A) UV kinetic traces of TPPS self-assembly at different concentrations of TPPS monomer and (B) a double-logarithmic plot of half-time ($t_{1/2}$) versus TPPS concentration.

Table S7. Global fitting parameters of UV kinetic traces

$[TPPS]_0$ (μM)	*A*	*B*	*m*	*n*	k_0 (s^{-1})	k_c ($M^{-1}s^{-1}$)	R^2
3	0.671	-0.397			1.90E-05	7.23E-04	1.00
4	0.719	-0.416	2.69	3.60	9.72E-05	9.65E-04	1.00
4.5	0.829	-0.444	(global)	(global)	1.68E-04	0.00127	1.00
5	0.936	-0.456			2.36E-04	0.00146	1.00

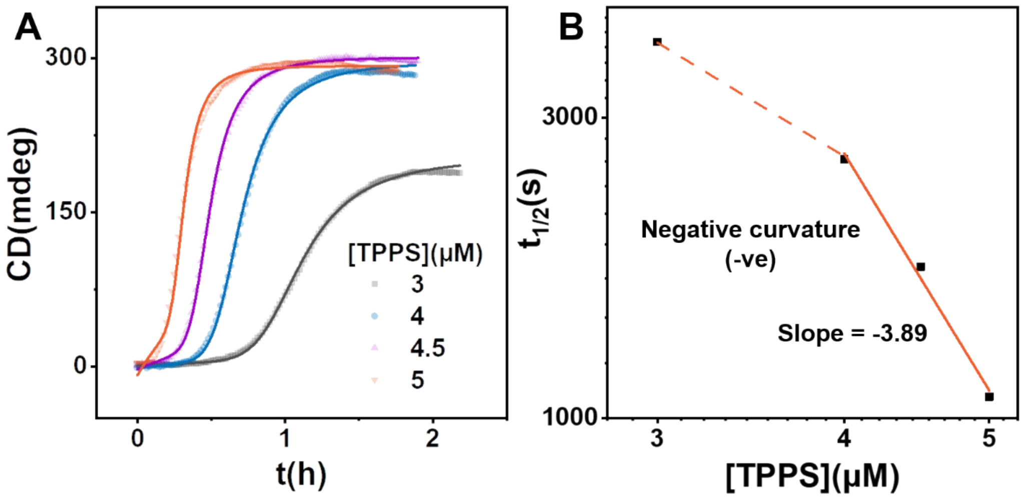

Figure S29. (A) CD kinetic traces of TPPS monomer assembly induced by 1.5 nM CMC at varying TPPS monomer concentrations and (B) the corresponding double-logarithmic plot of half-time versus TPPS concentration.

Table S8. Global fitting parameters of CD kinetic traces

$[TPPS]_0$ (μM)	*A*	*B*	*m*	*n*	k_0 (s^{-1})	k_c ($M^{-1}s^{-1}$)	R^2
3	-203	-204			1.04E-5	3.40E-4	1.00
4	-299	-297	3.02	7.00	1.48E-5	5.36E-4	1.00
4.5	-304	-301	(global)	(global)	5.47E-5	7.74E-4	1.00
5	-301	-292			2.29E-4	0.00123	1.00

The Double-logarithmic plots of half-time ($t_{1/2}$) versus monomer concentration, where the self-assembly process was monitored in real time using UV-Vis

spectroscopy, while the heterogeneous nucleator-mediated assembly was tracked using CD spectroscopy to minimize interference from homogeneous self-assembly.

From the double-logarithmic plots, we determined that the slope for homogeneous self-assembly is -1.31, while the slope for surface-catalyzed assembly is -3.89. Following the evaluation method described in the reference,[10] we further fitted the growth exponent $\boldsymbol{n}$ (corresponding to $\boldsymbol{n_c}$ in the reference). The analysis suggests that self-assembly is predominantly governed by primary nucleation and chain elongation ($-\frac{n_c-1}{2} < slop_{self} < -\frac{n_c}{2},\ n_c = 3.6,\ +ve$). In contrast, the heterogeneous nucleator-mediated assembly follows a mechanism dominated by nucleation, growth, fragmentation, and secondary nucleation ($-\frac{1}{2} < slop_{cat} < -\frac{n_c+1}{2},\ n_c = 7.0,\ -ve$). These results indicate significant mechanistic differences between homogeneous self-assembly and nucleator-induced assembly, demonstrating that the heterogeneous nucleator alters the assembly pathway from a nucleation-elongation model to a mechanism involving nucleation, growth, fragmentation, and secondary nucleation.

REFERENCES

1 L. Su, J. Xu, C. Lu, K. Gao, Y. Hu, C. Xue and X. Yan, *Cell Death Discov.*, 2024, **10**, 176.

2 R. F. Pasternack, C. Fleming, S. Herring, P. J. Collings, J. dePaula, G. DeCastro and E. J. Gibbs, *Biophys. J.*, 2000, **79**, 550-560.

3 R. F. Pasternack, E. J. Gibbs, P. J. Collings, J. C. dePaula, L. C. Turzo and A. Terracina, *J. Am. Chem. Soc.*, 1998, **120**, 5873-5878.

4 A. Weissman, H. Klimovsky, D. Harel, R. Ron, M. Oheim and A. Salomon, *Langmuir*, 2020, **36**, 844-851.

5 L. Kelbauskas, S. Bagdonas, W. Dietel and R. Rotomskis, *J. Lumin.*, 2003, **101**, 253-262.

6 N. Mataga, *Bull. Chem. Soc. Jpn.*, 1957, **30**, 375-379.

7 N. C. Maiti, M. Ravikanth, S. Mazumdar and N. Periasamy, *J. Phys. Chem.*, 2002, **99**, 17192-17197.

8 N. Micali, F. Mallamace, A. Romeo, R. Purrello and L. Monsù Scolaro, *J. Phys. Chem. B*, 2000, **104**, 5897-5904.

9 J. Kang, D. Miyajima, T. Mori, Y. Inoue, Y. Itoh and T. Aida, *Science*, 2015, **347**, 646-651.

10 G. Meisl, J. B. Kirkegaard, P. Arosio, T. C. Michaels, M. Vendruscolo, C. M. Dobson, S. Linse and T. P. Knowles, *Nat. Protoc.*, 2016, **11**, 252-272.
